# Defining and Measuring Organizational Transformation in Health Care: A Systematic Literature Review

**DOI:** 10.1177/10775587251356130

**Published:** 2025-08-13

**Authors:** Lauren Clack, Jason Smith, Martin Charns

**Affiliations:** 1 University of Zurich, Switzerland; 2 University Hospital of Zurich, Switzerland; 3 U.S. Department of Veterans Affairs, Boston, MA, USA; 4 Boston University, MA, USA

**Keywords:** organizational transformation, culture change, health care transformation, implementation science, quality improvement

## Abstract

Organizational transformation in health care is critical to achieving systemic improvements, yet it lacks a cohesive body of empirical literature. Thirty-six articles met inclusion criteria in this systematic literature review of empirical studies of whole-organization transformation describing the transformation process and measures of transformation. Studies had diverse analytic (*n* = 14) and descriptive (*n* = 22) aims and were published in many different journals. Few articles provided definitions of transformation. Most employed weak research designs, about half used models for evaluation, and no common measures of transformation were used across articles. Combinations of distributed leadership, staff engagement, and culture change were recurring themes contributing to successful transformation. Two-thirds of articles used models to guide the transformation process. There was no consistency across articles in which models were used for evaluating or guiding change. Most articles reported successful transformation. The literature is methodologically weak, highlighting the need for more rigorous, theory-driven research on health care transformation.

## Background

Understanding the dynamics of organizational transformation in health care is essential for guiding improvements in care delivery, yet critical knowledge gaps remain. The purpose of this systematic literature review is to assess what is known and what is not known about organizational transformation in health care. Health care organizations face increasing challenges related to aging patient populations, rising costs, national health care reforms, and shortages and burnout of health care professionals. There have been many calls to make health care organizations more patient-centered, safe, equitable, accessible, effective and efficient (Institute of Medicine (US) Committee on Quality of Health Care in America, 2001). Despite the calls for change, national policy and structural reforms, and interventions to improve individual processes, there has not been adequate progress. Some reasons for the slow progress are that health care organizations face substantial challenges related to their complex nature, interprofessional and interdisciplinary collaboration required by transformation initiatives, power differentials across professional groups and specialties, and consideration of the interests of patients, families, and communities (Institute of Medicine (US) Committee on Quality of Health Care in America, 2001). Organizational transformation may offer solutions to these challenges (Best et al., 2012; Garritsen et al., 2024; Lee et al., 2013). This article provides an updated comprehensive review of existing empirical studies of health care organizational transformation to assess whether gaps have been addressed since previous reviews were conducted. It takes a rigorous approach to examining how organizational transformation has been studied, measured, and evaluated empirically. By analyzing the research methods, measurement approaches, and reported outcomes, we aim to offer new insights into the mechanisms of transformational change and identify areas where further investigation is needed to advance both research and practice in health care settings.

### Why Transformation Is Needed

Most efforts to address the challenges in health care have focused on improvements of individual processes or parts of an organization (e.g., emergency department), as opposed to whole institution or (multi-) system level. The fields of implementation science and quality improvement both focus primarily on individual projects or parts of the organization. While these literatures recognize the importance of context to the success of interventions (Damschroder et al., 2009; Kaplan et al., 2012), they do not generally address improving the organizational context—that is, transforming the organization—to support interventions. Furthermore, although such interventions can be effective in improving specific aspects of care, their impacts remain isolated, seldom sustained, and often fail to address broader underlying barriers to delivery of high-quality care delivery (Al-Amin et al., 2024; Charns et al., 2022). Efforts at improving individual processes or parts of the organization are insufficient.

In contrast, transformation includes changing organizational culture—the collective values, norms, and assumptions that influence thought and behavior, as these are pivotal to how an organization functions (Sminia, 2016) and therefore more likely to lead to sustained changes (Hung et al., 2022). A *transformational* approach is defined as, “a multidimensional, multi-level, radical organizational change involving a paradigmatic shift” (Levy & Merry, 1986) that involves a sustained, organization-wide shift in patient care processes (Lukas et al., 2007) and “. . . departs radically from an organization’s past precedents, aims at large-scale readjustments, and is complex and systemic” (Lee et al., 2013).

Many approaches to organizational transformation in health care stem from other industries and employ performance improvement approaches, such as *lean management* (Toussaint & Adams, 2015), *six sigma* (Antony & Banuelas, 2002; Henderson & Evans, 2000), and principles of *high reliability organizations* (Roberts, 1990; Weick & Sutcliffe, 2007). These approaches involve cultivating an organizational culture that empowers staff to identify and address issues, improving processes through reduction of defects, and enhancing reliability through removal of errors while promoting an environment of “collective mindfulness,” respectively. While these have been described as supporting “whole system changes” and are intended to fundamentally change the culture and functioning of whole organizations, few transformations in health care have been reported in detail (Hung et al., 2022).

## New Contribution

There have been three reviews of organizational transformation in health care since 2012 (Best et al., 2012; Garritsen et al., 2024; Lee et al., 2013) and a related, narrower review specifically on change management (Harrison et al., 2021). The (Best et al., 2012) and (Lee et al., 2013) reviews are over a decade old. Lee et al. (2013) examined antecedents, processes (or paths) and outcomes of transformational change. They noted then, “. . . that organizational transformation in health care and other industries is a nascent research interest and that there is much room for research on past and current change initiatives,” and that conceptual and prescriptive papers outnumbered the identified empirical studies, suggesting that research on organizational transformation was still in a burgeoning phase. Consequently, we expect an updated review to identify an increased number of empirical studies on this topic. In a realist review commissioned by the Saskatchewan Canada Ministry of Health, Best et al. (2012) focused on the role of government and policy rather than on processes of transformation. Garritsen et al. (2024) used the Pawson and Tilley (1997) theory of change to examine interactions among context (“social, political, economic and historical circumstances or conditions”), mechanisms of change, and outcomes. Rather than describing intra-organizational dynamics, they focused primarily on inter-organizational collaboration across health care sectors. Harrison et al. (2021) reviewed change management models that were not necessarily models of transformation, and their search terms did not include any form of “transform.” Thus, while of interest and related to the topic of transformation, the Harrison et al. (2021) review is not directly on transformation.

In addition to the need for an updated review, prior reviews identified four gaps in the literature. First, there is no consensus on the conceptualization and definition of organizational transformation, and few articles even include a definition. Garritsen et al. (2024) found that only four of the 19 articles included in their review provided definitions of care transformation. The three other review articles did not report on whether articles in their review contained definitions of transformation. However, Best et al. (2012) stated, “An evidence base . . . is urgently needed for large-system transformation (LST), as there is no agreed-on definition of LST in the literature.”

Second, there is no consolidated source of information on how transformation has been studied, evaluated, and measured, nor about the evaluation models employed. Lee et al. (2013) noted the field still has important gaps in understanding how to measure organizational transformation and its success. Best et al. (2012) commented, “Evaluation demands a careful blending of quantitative measures and accountability with qualitative methods such as interviews, ethnographic observation, and storytelling to make sense of the transformation effort.” Lacking in the literature, however, is information on the prevalence of different study designs and methods. Most studies included in these reviews did not include direct measures of transformation itself, instead reporting more distal clinical or organizational outcomes that might result from transformation. They also did not report on what conceptual models were employed to evaluate transformation in their included articles. Furthermore, lacking in prior reviews is whether articles are descriptive or analytical, following Pettigrew’s (1985) suggestion for judging research articles.

Third, there is insufficient information on the processes/dynamics of transformational change and the models used to guide transformation. Best et al. (2012) reported there is an abundance of literature on antecedents or factors that influence transformation, but much less on actions undertaken to achieve transformational change or how these actions lead to observed results. Recognizing that health care organizations are complex adaptive systems and that it is unproductive to prescribe “action X to achieve outcome Y,” Best et al. (2012) identified “five simple rules” to guide transformation. They noted, however, their review was limited to what is reported in the literature itself and that the five simple rules may not be exhaustive. Lee et al. (2013) noted, “Few of the reviewed studies characterized the overall process of transformation or described it in sufficient detail.” In their review of change management methodologies, Harrison et al. (2021) noted that change management models were often used as “guiding framework . . . in keeping with contemporary thinking regarding health care as a complex adaptive system.” They also noted, “ . . . it was not possible to detect whether the use of a model, method or process contributed to the success.” Previous reviews have not synthesized reported conclusions about the factors contributing to transformation success, or lack thereof.

Fourth, there is little information on the proportion of transformation efforts that have been successful. Lee et al. (2013) noted there are gaps in the extent to which transformation is documented in the literature and furthermore that

. . . existing research literature may reflect a bias toward studies of successful transformation. This bias may in part be the result of difficulties in gaining access to organizations in which transformation initiatives stalled or failed. Lack of sufficient examination of unsuccessful initiatives raises concern about the validity of inferences about observed antecedents and consequences of transformational change. A positive publication bias may also stem from a tendency of researchers to define transformations by outcomes, such as financial turnaround, rather than by an initiative’s vision and goals.

Whether there is bias toward reporting successful transformations has not been well investigated.

To provide an updated review and address the gaps identified in the extant literature, this article aims to offer a comprehensive, systematic review of the empirical literature on organization-wide transformation in health care and to address the following specific research questions:

1. What are general characteristics of the literature on organizational transformation in health care?
2. How has organizational transformation in health care been conceptualized and defined?
3. How has organizational transformation been studied and measured? What conceptual models have been used to *evaluate* transformation?
4. What does the literature report on the dynamics of transformation and factors important to successful transformation? What conceptual models have been used to *guide* transformation efforts?
5. To what extent have transformation efforts in health care as represented in the literature been successful?

## Method

This systematic literature review followed Preferred Reporting Items for Systematic Reviews (PRISMA, http://www.prisma-statement.org) guidelines and was pre-registered with the PROSPERO database (PROSPERO 2020 CRD42020144573). We used Covidence Systematic Review Software for article screening, full-text review, and data extraction, and MaxQDA for coding text passages.

### Literature Search

Drawing on the expertise of our research librarian co-author (JS), we crafted a search strategy using a combination of available medical subject headings (MeSH, Emtree) and keywords. We searched peer-reviewed literature using PubMed, Embase, and Business Source Complete for English-language studies published from the start of the respective database records through January 13, 2023. Searches included a combination of terms related to transformation (e.g., organizational transformation, strategic organizational change, large system transformation) and health care settings. The complete search strategy is in Supplemental File 1. Search terms were informed by prior literature on organizational transformation in health care and were iteratively piloted and refined to ensure they captured known key articles of interest. Next, we searched the reference lists of included articles to identify additional articles.

### Inclusion and Exclusion Criteria

In line with our definition of organizational transformation, we included empirical articles that reported on whole organization transformation and excluded those focusing on specific processes or departments or deemed to be too narrow to be fundamental, organization-wide efforts. To address gaps in understanding of settings beyond acute care, we included all health care settings. We included both quantitative and qualitative study designs to gather a holistic perspective about “how” and “why” organizational transformation efforts perform. We excluded articles that did not report qualitative or quantitative outcomes or measures of transformation, and those that failed to describe the transformation process in sufficient detail.

### Study Selection Process

After removal of duplicates, two reviewers (LC, MC) independently screened all titles and abstracts. If inclusion and exclusion criteria could not be assessed conclusively during this screening, article full texts were retained and reviewed by the two reviewers. Disagreements were resolved through discussion to reach consensus.

### Data Extraction and Analysis

We identified and extracted key information from each article (authors, year of publication, journal, definition of transformation, study aims, description of transformation, study design, methods, measures, number of sites, setting, country, length of time for transformation, conceptual models mentioned, extent of success, and the full texts of qualitative results and conclusions of articles) into Covidence, then downloaded the complete database into MS Excel and MaxQDA for analyses. We examined the study aims, change and evaluation models, study design, measures, and success by creating pivot tables of these fields with other study characteristics.

### RQ1: General Characteristics of the Literature on Organizational Transformation

We categorized each article based on its aims into either “analytic” or “descriptive.” Analytic articles aimed to “explain,” “explore,” “understand,” “identify,” or “analyze” factors related to transformation, whereas descriptive articles aimed to “describe,” “document,” or “report.” We categorized the relation of authors to the studied organization(s) as internal (all authors employed by the organization), external (no authors employed by the organization) or both (internal and external authors). We considered consultants and external evaluators as external. We classified sites comprised of multiple entities as one site if there was a single management team for all sites (e.g., two hospitals comprising a trust) or if the transformation was of a whole multi-institutional system and data were reported only for the whole system. We also grouped articles by type of journal (e.g., health services research, health care management, clinical); however, we did not group *Implementation Science* with other journals and report it as a single journal.

### RQ2: How Has Organizational Transformation Been Conceptualized and Defined?

In each article, we recorded whether it mentioned transformation or defined transformation and extracted any definition of transformation provided.

### RQ3: How Has Organizational Transformation Been Studied and Measured?

We determined whether a study was qualitative or quantitative based on the methods reported in the article. Qualitative articles reported using interviews, focus groups, or observations. Quantitative articles reported surveys of staff, quantitative measures of quality, patient safety, or organizational performance. Articles using both qualitative and quantitative methods were classified as mixed methods. For all studies, to avoid ambiguities in terminology describing study design, we classified three features of the employed study design according to Higgins et al. (2013): inclusion of controls or comparisons, measurement of transformation before and after the intervention, site selection based on measures. We recorded conceptual theories, models, and frameworks used for evaluation of transformation, if any, and examined whether these models were used in multiple articles.

### RQ4: What Are Dynamics and Factors Important to Organizational Transformation?

Data extracted as qualitative results and conclusions about transformation were analyzed through a directed qualitative content analysis using the Organizational Transformation Model (OTM) (Charns et al., 2022; Lukas et al., 2007) as a deductive coding framework; topics not covered by the model were coded inductively. We chose the OTM because it is a model of organization-wide transformation specific to health care and includes specific factors reported to be associated with successful transformation. Some of the OTM constructs were specific to Lean Management Systems. In coding, we interpreted constructs broadly (e.g., for “Improvement projects” we considered all types of projects that involved staff). Following initial coding of all articles, similar codes were inductively grouped into thematic categories based on consensus discussion among two researchers (LC, MC). As we did for models used for evaluation, we recorded conceptual theories, models, and frameworks used for guiding the transformation change process, if any, and examined whether these models were used in multiple articles.

### RQ5: To What Extent Have Transformation Efforts in Health Care as Represented in the Literature Been Successful?

We categorized success of transformation as “yes” if authors described the intervention as successful in all sites, “no” if unsuccessful in all sites, “partial” if limited success in a single site or “varied” if success varied across sites in multi-site studies. To examine bias in reporting success, we used pivot tables to compare the success of transformation by article characteristics including analytic versus descriptive aims, use of models for evaluation and change, and authorship.

## Results

**Figure 1.**
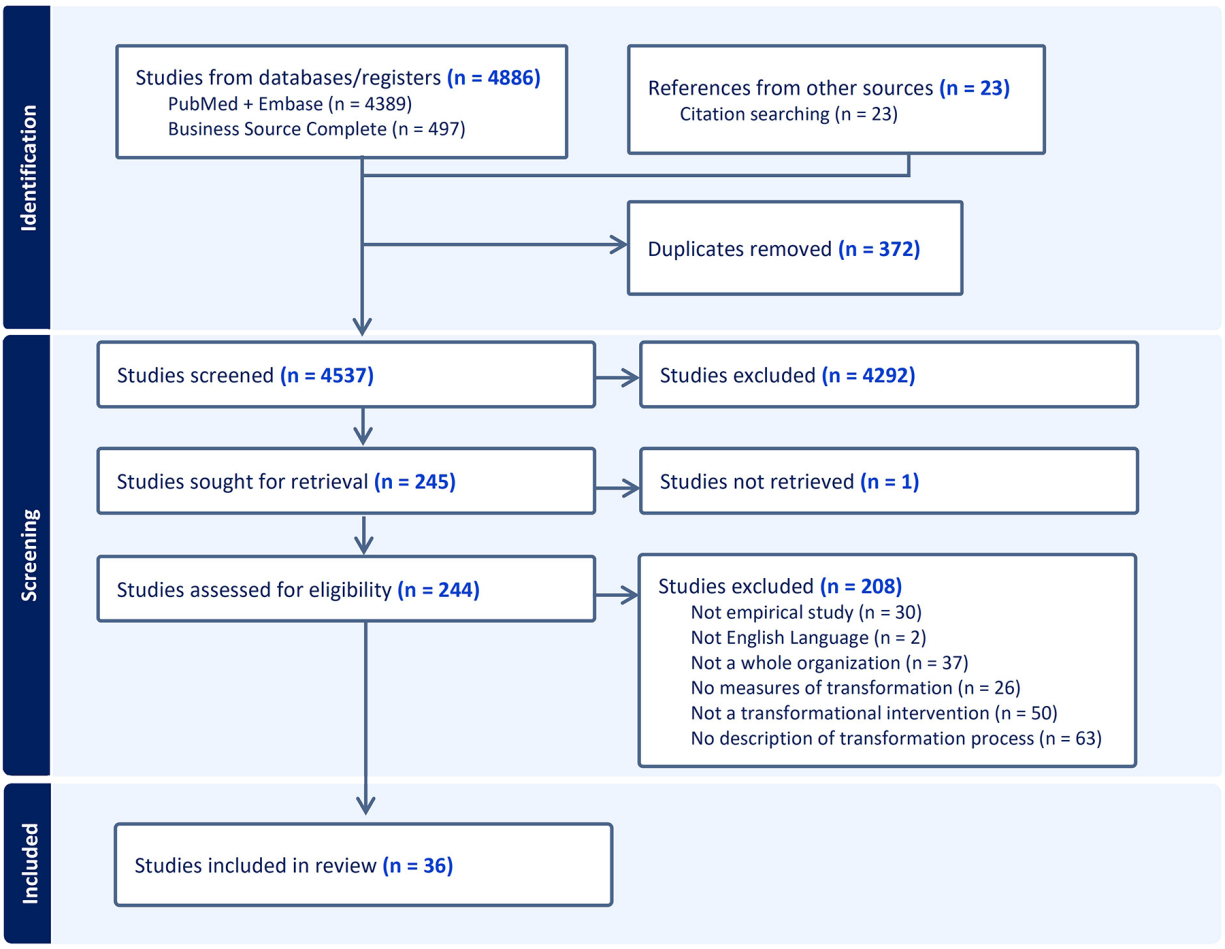
PRISMA Flowchart of Included Studies.

After removal of duplicates, we identified 4,537 unique articles and retained 244 during title and abstract screening. Following full-text review, 36 articles met inclusion criteria and were included in this review (Figure 1). Detailed information on each included article is shown in Table 1. Supplementary File 2 contains distributions of article characteristics (e.g., setting) and crosstabulations of characteristics derived from the pivot table analyses. Here, we highlight the most salient findings for each of our RQs.

**Table 1.** Characteristics of Included Articles.

Author (year) Title Journal	Definition of transformation	Name & description of intervention Study aims	Study design - C: Includes controls, comparisons, neither -M: Measurement of transformation before and after intervention -S: Site selection based on measure(s) Number and type of sites Country Length of time for transformation	Evaluation methods (research approach & each method of data collection) * Indicates article reports these data used to assess extent of transformation	Models: Mentioned as Background (B) Used as framework for Evaluation (E) Used to guide Change (C)	Extent of success: Yes Partial No Varied (across sites)	Conclusions about transformation
Azeem et al. (2015) Restraint Reduction at a Pediatric Psychiatric Hospital: A Ten-Year Journey. Journal of Child and Adolescent Psychiatric Nursing	Transformation not explicitly mentioned. Refers to culture change and introduction of evidence-based treatment modalities.	Intervention: Quality improvement Aims: Reduce use of restraints [Descriptive]	**C:** Neither **M:** Yes **S:** No 1 hospital US 10 years	Internal authors’ assessment **Interviews:** None reported **Focus Groups:** None reported **Surveys:** Survey of youths [clients] (No additional information reported) Staff surveys to monitor satisfaction and training needs (No additional information reported) **Other:** Use of restraints*	**B:** Restraint reduction **E:** None reported **C:** None reported	Yes. Based on reduction in use of restraints and authors’ report of positive culture change	Alignment, Capability Development, Community/ patient engagement, Culture, Intentional change process, Leadership, Monitoring and evaluation, Multiple factors, Organization and structure, Staff engagement
Barba et al. (2021) Data-driven quality improvement, culture change, and the high reliability journey International Journal of Healthcare Management	Transformation not explicitly mentioned. Refers to movement toward “high-reliability organization” as “. . . an ongoing process . . . that . . . might involve considerable culture change.”	Intervention: Shifting the hospital to a high-reliability organization (HRO). Aims: “. . . describe a transition journey undergone by a special hospital for those with developmental disabilities to a data-driven quality improvement and patient safety culture on a high reliability journey to deliver exceptionally safe, high quality care to every patient, every day.” [Descriptive]	**C:** Neither **M:** Yes **S:** No 1 hospital for people with medically complex developmental disabilities US 3.5 years	Senior leaders’ assessment of change based on staff surveys and quality data. **Interviews:** None reported **Focus Groups:** None reported **Surveys:** Employee workplace/engagement (Wave 1 n = 482, wave 2 n = 317)* AHRQ Surveys of Patient Safety Culture (SOPS) (Wave 1 n = 315, Wave 2 n = 316, Wave 3 n = 321]* Other: Organizational quality score card, including number of reports of patient safety events and medication errors.	**B:** AHRQ CUSP model, High reliability organizations (HRO) **E:** None reported **C:** None reported	No. “Despite favorable trends in quality and patient safety data . . . achieving full ‘buy-in’ . . . from all staff is a continuing challenge.”	Communication, Culture, Leadership, Monitoring and evaluation, Staff engagement
Bate et al. (2000) Towards a Culturally Sensitive Approach To Organization Structuring: Where Organization Design Meets Organizational Development. Organization Science	Transformation mentioned, but not explicitly defined. Refers to second order change (Levy & Merry, 1986) and “ . . . cultural change: a re-evaluation of the way people thought and felt about their work and their professional relations with others, and a restoration of the psychological and emotional contract between the individual and the organization.”	Intervention: Action research using organization development and organization design Aims: Determine (1) “how leaders and change agents can actually begin to intervene in and manage the complex dynamics of cultural change” (2) explore the relationship between culture and structure in the context of a “culturally sensitive restructuring” of a hospital trust [Analytic]	**C:** Neither **M:** Yes **S:** No 1 Trust with 3 hospitals (Data presented for Trust as a whole) UK 2 years	External organization development consultants (authors) assessment of changes in culture **Interviews:** Consulting physicians, managers, nurses and ancillary staff (n not reported)* Focus Groups: Consulting physicians, managers, nurses and ancillary staff (n not reported)* Surveys: None reported **Other:** “observational loitering,” participant observation of key meetings, feedback and planning sessions. 150 visits and 500 days in the field*	**B:** Cultural dynamics, Organization design (designing), Leadership “realized in the process whereby one or more individuals succeeds in attempting to frame and define the reality of others” **E:** Action research **C:** Action research, Culturally sensitive restructuring (CSR)—Model specific to this study	Yes. “The organization was able to agree on a new design, to realize a cultural change, and to prime itself for a major relocation.”	Culture, Impetus for change, Leadership, Multiple factors, Organization & structure, Organizational learning, Staff engagement
Benning et al. (2011) Large scale organisational intervention to improve patient safety in four UK hospitals: mixed method evaluation. British Medical Journal (BMJ)	Transformation not explicitly mentioned. Refers to “. . . avoid unnecessary harm, pain or suffering as a result of error in medical intervention.”	Intervention: Safer Patient Initiative—Quality Improvement Aims: To conduct an independent evaluation of the first phase of the Health Foundation’s Safer Patients Initiative (SPI) [Descriptive]	**C:** Controls **M:** Yes **S:** Not reported 22 hospitals – 4 intervention & 18 control UK 1.5 years	External evaluation with 3 rounds of data collection **Interviews:** Interviews with strategic/senior staff (n = 60) and with staff on wards (n = 88) **Focus Groups:** Focus groups in acute wards (n = 4/site; n of individuals not reported) **Surveys:** National NHS staff survey of satisfaction, morale and organizational climate (Wave 1 control sites n≈3,600; wave 2 control sites n≈3,600; wave 1 intervention sites n≈3,900; wave 2 intervention sites n≈3,900)* NHS Patient surveys **Other:** Observations of compliance in taking observations of patients, Consumption of soap and alcohol hand rub, Case note review to identify errors and adverse events, Patient outcomes	**B:** None reported **E:** None reported **C:** Institute for Healthcare Improvement (IHI)	No. Based on only 1 of 11 changes in survey measures favoring SPI1 hospitals over controls and concerns about difficulties in implementing the program	Facilitation/consultation, Impetus for change, Intentional change process, Leadership, Staff engagement, Time
Bess et al. (2009) Participatory organizational change in community-based health and human services: from tokenism to political engagement. American Journal of Community Psychology	Transformation mentioned, but not explicitly defined. They aimed to achieve change in the “organizations’ managerial and practice paradigm from one based on first-order, ameliorative change to one that promotes second-order, transformative change via strength-based approaches, primary prevention, empowerment and participation and focuses on changing community conditions.”	Intervention: Action research to promote strength-based practices, prevention, empowerment, and community condition change (SPEC) Aims: “. . . document factors that facilitate or inhibit meaningful participation of staff and community members in the promotion of personal, relational, and collective well-being of workers and citizens alike.” [Analytic]	**C:** Neither **M:** No **S:** No 2 community-based health and human service organizations US 2 years	External authors’ assessment of the integration of “SPEC principles” (not explicitly defined) into the respective organizations **Interviews:** key informants representing a cross-section of the organization with different organizational roles and “snowballed” participants (n = 15)* Focus groups: 3–10 organizational members, community members & researchers per focus group (n = 10)* Surveys: None reported **Other:** Participant observations of meetings & events* Review of artifacts (meeting agendas, team products, program related information, organizational promotional materials, and project related correspondence)*	**B:** Participatory processes involving community members or recipients of services, Ameliorative paradigm **E:** Action research **C:** Action research	No. “The move toward political engagement in this project has been slow but continues at [one site]. . . . At [other site] most T-team members never moved beyond precontemplation as a group and participation never got beyond tokenism.”	Alignment, Community/patient engagement, Culture, Leadership, Organizational learning, Staff engagement
Best et al. (1997) Healthy hospital: toward a better tomorrow. A demonstration project to promote culture change through participatory decision making. Healthcare Management Forum	Transformation not explicitly mentioned. Refers to culture change and improving job satisfaction through participatory decision-making.	Intervention: “Healthy Hospital” demonstration project. “[P]roject designed to increase participatory decision making for all staff groups, thereby decreasing job stress and increasing job satisfaction.” Aims: “ . . . demonstrate ways to change culture around participatory decision-making focus, document lessons learned throughout the change process, and measure the effects of these innovations.” [Descriptive]	**C:** Neither **M:** Yes **S:** No 1 hospital Canada 3 years	Internal and external authors’ assessment of change. **Interviews:** 2 waves with each member of project steering committee (n not reported)* Focus groups: 2 waves with staff (n not reported)* Surveys: 3 waves of surveys of staff and management to assess job stress, job satisfaction, work climate, self-esteem, intent to stay at the hospital, psychological job demand, decision latitude, social support, hazardous exposure (Wave 1 n = 280, wave 2 n = 319, wave 3 n = 247)* Other: None reported	**B:** Quality of worklife **E:** Model specific to this study **C:** None reported	No. After initial gains “ . . . comparison of the 1993 to 1994 data suggests either . . . little further improvement or a partial loss of the 1991-1993 gains.”	Alignment, Capability Development, Communication, Facilitation/ consultation, Goals, Intentional change process, Leadership, Monitoring and evaluation, Multiple factors, Organization and structure, Organizational learning, Staff engagement, Sustainment, Time
Brooks (1996) Leadership of a cultural change process. Leadership & Organization Development Journal	Transformation mentioned, but not explicitly defined. Refers to “turnaround” and “significant cultural change to an organization . . .”	Intervention: CEO and management interventions to reorganize and gain trust status Aims: “To explore the successful role of leadership in initiating and sustaining a major change process . . .” [Analytic]	**C:** Neither **M:** No **S:** No 1 hospital UK 3 years	External author’s evaluation. **Interviews:** Staff at various levels (n = 20) **Focus Groups:** None reported **Surveys:** None reported **Other:** Observation of meetings, Author’s report of consultants’ (specialty physicians) decisions to accept change.* Achievement of trust status.*	**B:** None reported **E:** None reported **C:** Model specific to study	Yes. Changes needed to achieve trust status were achieved.	Alignment, Communication, Community/patient engagement, Culture, Goals, Impetus for change, Intentional change process, Leadership, Monitoring and evaluation, Organization and structure, Organizational learning, Staff engagement
Centauri et al. (2018) System-wide lean implementation in health care: A multiple case study Health Services Management Research	Transformation mentioned, but not explicitly defined Refers to implementation of “lean transformation” organization-wide and “a holistic approach . . . in which long-term improvement goals are aligned to the overall organizational strategy, which in turn leads to lean practices to be broadly embraced at all levels of the organizations.”	Intervention: Lean transformation Aims: To develop a “deeper understanding of how lean interventions interact with the organizational context . . . in order to make lean spread and stay as an organizational-wide approach.” [Analytic]	**C:** Neither **M:** No **S:** Yes 3 hospitals Italy Varied by hospital: 7–10 years	External evaluation by authors. Three sites identified for study through national survey of Lean implementation conducted prior to study. **Interviews:** n = 18 (4–8 per site) with key lean individuals and frontline professionals who had an active role in lean **Focus Groups:** None reported **Surveys:** National survey of staff in Italian hospitals—no additional information reported* Other: Internal document review, Review of published articles and books on the sites*	**B:** Socio-technical systems **E:** Model specific to study based on Socio-technical systems **C:** Lean	Yes. All 3 sites selected on the basis of their high level of diffusion and sustainment of Lean practices as determined by national survey of Italian hospitals.	Alignment, Capability Development, Leadership, Monitoring and evaluation, Multiple factors, Organization and structure, Organizational learning, Staff engagement, Sustainment
Charns et al. (2022) Achieving transformation to lean management systems in health care Health Services Research	Lean transformation: “Lean thinking, a quality improvement (QI) philosophy supported by concepts, methods, and tools . . . Lean encourages continuous QI through methodical visualization-driven process improvement and standardization . . .” Fostering a culture of	Intervention: Lean Management Systems Aims: “To understand what factors and organizational dynamics enable Lean transformation of health care organizations” [Analytic]	**C:** Comparisons **M:** Yes **S:** No 7 hospitals US 3 years	External evaluation by authors **Interviews:** 98 interviews with n = 121 individual participants, executive leaders, middle management, value stream owners, frontline staff, senseis* Focus Groups: Frontline staff and supervisors* (N’s reported together with individual interviews) **Surveys:** None reported	**B:** Organization Transformation Model (OTM) **E:** Model developed in this study based on markers of transformation, OTM **C:** Lean	Varied. Sites classified into high, medium and low transformation based on a transformation score computed from ratings of qualitative data.	Alignment, Capability Development, Communication, Community/patient engagement, Impetus for change, Leadership, Monitoring and evaluation, Multiple factors
	continuous improvement is essential.			**Other:** Transformation score computed as sum of products of ratings of depth & spread of 11 prospectively defined markers of transformation based on data from interviews, focus groups & review of reports & presentations*			
Cohen et al. (2004) Implementing a hospitalwide patient safety program for cultural change. Joint Commission Journal on Quality & Patient Safety	Transformation not explicitly mentioned. Refers to culture change: “ . . . establish a more effective culture of patient safety in the hospital, as defined by ‘the willingness of all staff members to report all safety events and near misses without fear of retribution, but with an understanding of accountability.’”	Intervention: Introduction of a patient safety program. Aims: describe “. . . the development and implementation of a comprehensive patient safety program for establishing a culture of patient safety in a community hospital.” [Descriptive]	**C:** Neither **M:** Yes **S:** No 1 hospital US 3 years	Internal authors assessment of transformation. **Interviews:** None reported **Focus groups:** 6 focus groups with physicians, nursing staff, unit clerks and pharmacy staff (n = 68 individuals) **Surveys:** A sample of staff were surveyed monthly on staff satisfaction and patient safety (n = 32 on average)* Other: Patient safety event reporting: medical events, medication events and phone calls to hotline*	**B:** Just culture, IoM To Err is Human, Joint Commission Accreditation Manual **E:** None reported **C:** None reported	Yes. Significant increases in event reporting, hotline calls, rate of serious medication events, and “ . . . staff awareness of importance of patient safety & willingness to report safety events.”	Alignment, Communication, Community/patient engagement, Culture, Facilitation/consultation, Leadership, Multiple factors, Organization and structure, Sustainment
Connor et al. (2007) Creating a fair and just culture: one institution’s pat toward organizational change. Joint Commission Journal on Quality & Patient Safety	Transformation mentioned, but not explicitly defined. Refers to creating a fair and just culture.	Intervention: Implement a patient safety program. Aims: “ . . . describe the development and implementation of . . . principles [of fair and just culture] and the transformation under way at DFCI toward a culture that is fair and just.” [Descriptive]	**C:** Neither **M:** Yes **S:** No 1 cancer institute US 5 years	Internal authors’ assessment of transformation. **Interviews:** None reported. **Focus groups:** None reported. **Surveys:** Staff survey of satisfaction and respect at 2 time points (n not reported)* Other: None reported	**B:** Just culture, IoM To Err is Human **E:** None reported **C:** None reported	Yes. Based on sustainment of improved survey scores in clinical areas and improvement in non-clinical areas	Capability Development, Culture, Leadership, Multiple factors, Staff engagement
Corring et al. (2016) Mental Health System Transformation: Drivers for Change, Organizational Preparation, Engaging Partners and Outcomes Healthcare Quarterly	Transformation mentioned, but not explicitly defined. Focus on culture change from institutional care to “recovery-oriented care”	Intervention: Achieve recovery-oriented service delivery through multiple strategies including “. . . engaging stakeholder groups in developing an action plan; . . . focusing on building a culture of affirmation, respect and hope; using recovery-oriented language in care planning and documentation; . . . involving community partners . . .” Aims: No research aim stated. [Descriptive]	**C:** Neither **M:** No **S:** No 1 hospital Canada 9 years	Internal authors’ assessment of transformation **Interviews:** None reported. **Focus groups:** Focus groups with staff (n not reported) **Surveys:** Patient satisfaction survey (n not reported)* Other: None reported	**B:** Recovery-oriented care **E:** None reported **C:** None reported	Partial. Significant progress has been made, but we are not done yet . . .	Capability Development, Communication, Facilitation/consultation, Leadership, Time
Foster (2016) Improving organisational culture through quality improvement, values-based leadership and staff engagement: An NHS trust case study. Management In Health Care	Transformation not explicitly mentioned. Refers to changing culture.	Intervention: “Go engage” Staff Engagement Pathway Model and IHI Quality improvement model Aims: “. . . to share the approach taken at WWL to improve organisational culture change through a focus on quality improvement and staff engagement.” [Descriptive]	**C:** Neither **M:** Yes **S:** No 1 trust with 3 facilities (Data presented for Trust as a whole) UK 15 years	Chief executive reports on transformation. **Interviews:** None reported **Focus Groups:** None reported **Surveys:** Annual Picker NHS national staff engagement survey and quarterly internal staff engagement survey (n’s not reported)* Other: External awards, Sickness absence, Clinical outcomes (reduction in harms, mortality), Financial savings	**B:** IHI, Langley et al. Improvement Guide **E:** None reported **C:** IHI, Langley et al. Improvement Guide, WWL wheel—model specific to this study	Yes. Change in culture primarily measured as staff engagement.	Alignment, Capability Development, Culture, Goals, Leadership, Monitoring and evaluation, Staff engagement, Time
Golden-Biddle (2020) Discovery as an Abductive Mechanism for Reorienting Habits within Organizational Change Academy of Management Journal	Transformation mentioned, but not explicitly defined. Mentions “organizational change in the form of a new care delivery model”	Intervention: New model of “Collaborative” inpatient care delivery, which embraced “. . . a vision of hospital care with nursing at its center provided in an environment designed specifically . . . to ensure safety and to promote healing.”.	**C:** Neither **M:** No **S:** Yes 1 healthcare system with 4 hospitals & 20 primary care practices US 9 years	External author assessment **Interviews:** (n = 30, 25 initial and 5 follow-up) with clinicians, managers, staff members, and senior leaders involved in creating the new care system*; Also informal, conversational interviews* Focus Groups: None Reported **Surveys:** None Reported	**B:** None reported **E:** Abduction **C:** Lean	Yes. “By 2013 all inpatient units were onstream.” “These units continued to show improvements in quality, patient satisfaction, and nursing satisfaction.”	Impetus for change
		Aims: “. . . to understand peoples lived experience of creating organizational change in the form of a new care delivery model.” [Analytic]		**Other:** Review of emails and phone calls, Review of documents,* Observations of meetings and units,* Site visits, Archival data “ . . . including nursing satisfaction surveys and compilation of comparative care metrics; reports and planning documents about the change initiative . . .”*			
Henry et al. (2017) Application of Kotter’s Theory of Change to Achieve Baby-Friendly Designation. Nursing for Women’s Health	Transformation not explicitly mentioned. Refers to culture change	Intervention: Apply Kotter’s Theory of Change to implement principles of baby friendly hospitals [Use of 10 steps to increasing breastfeeding] Aims: Achieve baby-friendly hospital designation [Descriptive]	**C:** Neither **M:** Yes **S:** No 1 hospital US 6 months	Assessment by external BFHI (Baby-friendly hospital initiative) surveyors. Internal authors’ report of transformation. **Interviews:** Staff, health care providers and women receiving care (n not reported) **Focus groups:** None reported **Surveys:** None reported **Other:** Observations of birth and breastfeeding practices Site visits by BFHI surveyors*	**B:** Baby-friendly initiative **E:** None reported **C:** Kotter’s change model	Yes. Surveyors assess culture change to support BFHI principles and hospital received BFHI certification.	Culture, Intentional change process
Hilliard et al. (2012) Our journey to zero: reducing serious safety events by over 70% through high-reliability techniques and workforce engagement. Journal of Healthcare Risk Management	Transformation mentioned, but not explicitly defined. Refers to transformation to a high-reliability safety culture.	Intervention: Safety Transformation Initiative (STI), high reliability techniques, power of one Aims: Report the results of the “Safety Transformation Initiative” to transform safety culture and reduce adverse events [Descriptive]	**C:** Neither **M:** Yes **S:** No 1 hospital US 5 years	Internal authors’ assessment of improvement in safety culture and decrease in adverse events **Interviews:** None reported **Focus groups:** None reported **Surveys:** Annual AHRQ safety culture, employee engagement surveys (n’s not reported)* Other: Adverse event reporting,* External awards,* Financial performance*	**B:** High reliability Organization, Just Culture, HPI Patient safety measurement system **E:** None reported **C:** None reported	Yes. Improvements in serious safety event rate (SSER), rate of event reporting, finances, safety culture survey. External recognition through awards.	Intentional change process, Staff engagement
Hunter et al. (2014) A mixed-methods evaluation of transformational change in NHS North East Health Services and Delivery Research	Transformation mentioned, but not explicitly defined. Refers to achieving a change in overall culture: “. . . create a NHS culture that encouraged innovation and success, and one that fostered a learning culture which made good use of best practice exemplars” quoting Department of Health (1998)	Intervention: North East Transformation System (NETS)—Improve care pathways, through Vision, Compact, Method; QI Aims: improving efficiency and effectiveness of care pathways for staff and patients; identification of the factors facilitating, and/or acting as barriers to, successful change. [Analytic]	**C:** Comparison **M:** No **S:** No 9 study sites in regional healthcare system: 2 clusters of primary care trusts, 2 mental health and learning disability trusts, 3 hospital trusts, ambulance trust and community services trust UK 3.5 years	External authors’ assessment based on qualitative data **Interviews:** Interviews with clinicians, managers, administrators, board members (n = 68)* Focus groups: 2 with Human Resources staff and managers* Surveys: None reported **Other:** 4 observation sessions (of RPIWs), Documentary analysis, Routinely collected NHS performance data	**B:** Lean, Toyota production system, Sociotechnical design **E:** Pettigrew et al. Receptive contexts for change, “Three-legged stool: Vision, Compact & Method”—specific to this study **C:** Virginia Mason Production System (VMPS)	Varied: “Progress was sustained at four of the study sites, but slowed or ceased at the other sites . . . The NETS . . . succeeded in bringing about real and lasting change in some parts of the North East. However, it was unable to fully realise its vision and purpose partly because of the widespread reorganisation of the NHS . . .”	Impetus for change, Leadership, Sustainment, Time
Jones et al. (2013) A theory-driven, longitudinal evaluation of the impact of team training on safety culture in 24 hospitals. BMJ Quality & Safety	Transformation mentioned, but not explicitly defined. Refers to implementation of safety culture “. . . defined as the learned, shared, enduring values and behaviours of organisation members regarding the organisation’s willingness to detect and learn from errors.”	Intervention: Team strategies and tools to enhance performance and patient safety (TeamSTEPPS) Aims: Evaluate the impact of a team training intervention on hospital safety culture [Descriptive]	**C:** Controls **M:** Yes (intervention hospitals only) **S:** No 37 critical access hospitals (24 intervention, 13 control) US 1 year	External authors’ report of comparison of intervention to control sites. **Interviews:** None reported **Focus Groups:** None reported **Surveys:** HSOPS survey of employees to assess safety culture, and items measuring extent of team training, learning and transfer (Baseline: intervention hospitals only, n not reported; post-intervention all 37 hospitals n = 3,465)* Other: None reported	**B:** Safety culture **E:** Diffusion of Innovation, Kirkpatrick & Kirkpatrick Evaluating training programs **C:** TeamSTEPPS	Yes. Intervention group HSOPS scores were significantly higher than static group scores in three dimensions assessing the flexible and learning components of safety culture.	Alignment, Capability Development, Culture, Leadership, Multiple factors, Staff engagement
Kerr et al. (2015) Changing organisational leadership culture: focus on values changes culture. Future Hospital Journal	Transformation not explicitly mentioned. Focus is on culture change and change in leadership and behaviors at all levels	Intervention: Distributed leadership through appreciative inquiry Aims: To collaboratively establish and then embed a values-based culture based on distributed leadership principles [Descriptive]	**C:** Comparison (of staff engagement survey scores to other NHS hospitals) **M:** Yes **S:** No 1 NHS Trust (2 hospitals) UK 9 years	Internal authors’ report based on comparison of percentage of positive scores pre- and post-intervention on NHS staff survey **Interviews:** None reported **Focus groups:** None reported **Surveys:** NHS employee survey (n not reported)* Other: Information from employee assessments	**B:** Distributed leadership **E:** None reported **C:** Appreciative inquiry, Values and behaviors framework—model specific to this study	Yes. Based on change in staff survey results. “Today 86% of our staff think care of patients is the organisations [sic] top priority and 85% would recommend us as a place to receive treatment”	Culture, Goals, Leadership, Multiple factors
Kirch et al. (2006) Reinventing the academic health center Academic Medicine	Transformation mentioned, but not explicitly defined.	Intervention: De-merger and creation of realigned college of medicine and medical center Aims: “We describe the sweeping transformation in an AHC [academic health center] and analyze the critical success factors that allowed this transformation to occur.” [Analytic]	**C:** Neither **M:** Yes **S:** No 1 academic health center (medical school & hospital) US 4 years	Internal authors’ assessment **Interviews:** None reported **Focus Groups:** None reported **Surveys:** Morale survey of staff, faculty & residents (n not reported)* Patient surveys (n not reported)* Other: Organizational performance measures on students, research funding, clinical encounters, fund-raising*	**B:** Mission-based management for re-aligning financial resources **E:** None reported **C:** None reported	Yes. “There is widespread belief within [the organization] that a fundamental organizational transformation has occurred.” “The outcomes measures . . . provide strong support . . . that the AHC . . . has been reinvented, with a resultant surge in organizational performance.”	Impetus for change, Multiple factors
Lord et al. (1998) Analysis of change within a mental health organization: A participatory process Psychiatric Rehabilitation Journal	Transformation mentioned, but not explicitly defined. Refers to “mental health reform” and associated “organisational change” and Carling’s (1995) roles that are consistent with the shift in mental health programs to an empowerment focus.	**Intervention:** Action research. Mental health reform incorporating “strategic planning” with elements of “transactive planning.” **Aims:** “. . . to document the process of change associated with a mental health organization, while offering a case study which included the context and motivation for change, planning and implementation, and eventual outcomes linked to the process of change.” [Descriptive]	**C:** Neither **M:** No **S:** No 1 community-based mental health organization Canada 3 years	Authors’ (including executive director and external evaluator and facilitator) assessment of extent of achievement of the goals set out in the strategic plan and more general assessment of extent of organizational change. **Interviews:** None reported **Focus Groups:** Focus groups of consumers and staff—no additional information reported* Surveys: Staff and consumer surveys—no additional information reported* Other: Document analysis: strategic plan, implementation strategy; Documents produced by external evaluator*	**B:** Resource mobilization theory, Three key values that distinguish traditional and emerging mental health paradigms, Learning Organization, Participation from stakeholders **E:** Carling’s Roles consistent with shift to an empowerment focus **C:** Transactive Planning	Yes. “The evaluation showed many positive results that reflected the changes at different levels of analysis.” Also cited culture change.	Alignment, Community/patient engagement, Culture, Organizational learning, Staff engagement
Lukas et al. (2007) Transformational change in health care systems: an organizational model. Health Care Management Review	“Transformational change, by contrast [to isolated QI efforts] is pervasive and involves not only structures and processes but also the inherent culture and values of the health care organization” (NHS Institute for Innovation and Improvement, Matrix Research and Consultancy, 2006)	Intervention: The Pursuing Perfection (P2) Program, which “sought to achieve dramatic improvements in patient outcomes by pursuing perfection in all major care processes . . .” Aims: To strive for perfect patient care, “moving organizations from short-term, isolated performance improvements to sustained, reliable, organization-wide, and evidence-based improvements in patient care.” “understanding the factors that contributed to (or impeded) the health care systems’ abilities to achieve their goals.” [Analytic]	**C:** Comparison **M:** Yes **S:** No 12 healthcare systems US 3.5 years	External authors’ assessment of extent to which systems displayed the 5 elements of the Organization Transformation Model **Interviews:** With CEOs, clinical executive staff, senior QI managers, interdisciplinary QI project team members, frontline staff, managers responsible for information technology, human resources, and other business functions (n = 750 over 7 waves of data collection)* Focus groups: Many interviews were in a group setting (n not reported separately from interviews)* Surveys: Not reported **Other:** Review of strategic plans, improvement team workplans, team and organizational performance measures, and communication materials.	**B:** IoM Crossing the Quality Chasm, Baldrige National Quality Program **E:** Complex organizational change, Diffusion of innovations, Microsystem effectiveness, OTM—Model specific to this study **C:** IHI	Varied. Sites had different extents of transformation: “ . . . most [sites] made substantial progress in improving clinical performance in targeted areas, and some made strides in redesigning systems to support broader organizational changes.”	Alignment, Capability Development, Goals, Impetus for change, Leadership, Multiple factors, Organization and structure, Staff engagement, Sustainment, Time
McKellar et al. (2020) Everyone matters; everyone contributes, everyone grows: a pilot project cultivating psychological safety to promote growth-oriented service culture after the Oakden Report Australian Health Review	Transformation mentioned, but not explicitly defined. The Oakden Report Response Plan presented a codesigned culture framework as a blueprint for service reform.	Intervention: Deliberatively developmental organization (DDO) for personal and professional growth Aims: Present a pilot. . . experientially exploring an innovative approach to health service culture change. [Descriptive]	**C:** Neither **M:** Yes **S:** No 1 Long-term care and mental health facility Australia 32 weeks	Internal authors’ self-report **Interviews:** Interviews by independent evaluation with team members in phase 1 (n not reported). **Focus Groups:** None reported **Surveys:** Staff survey conducted pre and post phase two to assess organizational climate and personal engagement in growth culture (n’s not reported)* Weekly pulse survey to assess team morale and engagement (n’s not reported)* Other: Frequency of incident reporting, Reduction in sick leave	**B:** Manley & Jackson Five features of effective workplace culture, Razzetti More productive meetings **E:** Psychological safety, Deliberately developmental organization **C:** None reported	Partial. “[The] transformation journey . . . is a work-in-progress.”	Capability Development, Community/patient engagement, Culture Facilitation/ consultation, Goals, Intentional change process, Leadership, Multiple factors, Organizational learning, Staff engagement, Sustainment
McNulty and Ferlie (2004) Process Transformation: Limitations to Radical Organizational Change within Public Service Organizations. Organization Studies	Refers to Tushman & Romanelli (1985) “. . . sharp and simultaneous shifts in strategy, distribution of organizational power, structure and control mechanisms”; Pettigrew’s (1985, 1987) conceptualization of “. . . strategic change and transformation as change in dominant ideologies, cultural systems of meaning and power relations”; and Greenwood & Hinings’ (1996) “archetype,” “a configuration of structures and systems of organizing with a common orientation or ‘underlying interpretative scheme.’”	Intervention: Reengineering Aims: “. . . describe and explain the introduction and impact of reengineering within a UK hospital.” “Specifically, the study sought to explore: how change took place or did not take place; who supported the change process and who resisted; and what underlying power resources were brought to bear to influence the outcomes of the change process.” [Analytic]	**C:** Neither **M:** No **S:** No 1 hospital UK 3 years	Authors’ external evaluation of the extent of changes in empirically observable patient services **Interviews:** Staff at various levels and disciplines (n = 144)* Focus Groups: None reported **Surveys:** None reported **Other:** Observations of meetings and informal conversations (n~50 individuals and processes),* Case studies of six clinical areas developed from Interviews and observations,* Economic analysis part of larger evaluation published as separate article, Document analysis of history of the hospital, transition to NHS Trust status, introduction of clinical directorates, earlier quality improvement initiatives & genesis & early development of the BPR program	**B:** Process Reengineering **E:** Pettigrew, Ferlie et al. Model for assessing organizational transformation, Understanding radical organizational change **C:** Process Reengineering	No. Overall, the change was limited and described as evolutionary rather than revolutionary.	Goals, Leadership, Organization and structure, Staff engagement
Ochocka et al. (1999) Organizational change towards the empowerment-community integration paradigm in community mental health. Canadian Journal of Community Mental Health	Transformation not explicitly mentioned. Refer to “. . . paradigm shift in the values and related practices within the organizations”	Intervention: Community empowerment through action research Aims: “to understand the experience of change as three mental health organizations strove to implement an empowerment community integration paradigm” [Analytic]	**C:** Neither **M:** No **S:** No 3 community mental health organizations Canada 2.5 years	External authors’ assessment of change in values **Interviews:** Executive directors of the 3 organizations (n = 3)* Focus groups: Stakeholders (managers, staff, consumers/survivors, family members, volunteers) of the 3 agencies (n = 85)* Surveys: None reported **Other:** Observations of meetings,* Review of background information	**B:** Social-constructivist paradigm **E:** Participatory-action research **C:** Participatory-action research	Yes. “The three organizations made substantial changes toward the values of the empowerment-community integration paradigm”	Alignment, Community/patient engagement, Goals, Staff engagement
Peirson et al. (2012) Building capacity for evidence informed decision making in public health: a case study of organizational change. BMC Public Health	Transformation mentioned, but not explicitly defined. Implicit in efforts to achieve evidence-informed decision making (EIDM) throughout the organization.	Intervention: Integrated knowledge translation (Authors involved in change process, similar to action research) Aims: “This research was conducted to explore and describe critical factors and dynamics in the early implementation of one public health unit’s strategic initiative to develop capacity to make EIDM standard practice.” [Analytic]	**C:** Neither **M:** No **S:** No 1 public health organization Canada 2 years	External & internal authors’ assessment of attitudes, organization and staff capacity for EIDM over time. **Interviews:** 2 rounds with public health unit participants (n = 4, n = 2)* Focus groups: Medical officer, associate medical officers, library personnel, and directors, managers, supervisors and specialists (n = 9, n = 12 focus groups and 64 individuals)* Surveys: None reported **Other:** 2 rounds of document review*	**B:** Evidence-based decision making in public health **E:** Stages in the evidence-informed public health process **C:** Integrated knowledge translation	No. “. . . staff continued to struggle with key barriers . . . ”	Alignment, Capability Development, Communication, Culture, Goals, Leadership, Monitoring and evaluation, Multiple factors, Organization and structure, Organizational learning
Peterson et al. (2012) A safety culture transformation: its effects at a children’s hospital Journal of Patient Safety	Transformation mentioned, but not explicitly defined. Refers to change in safety culture, use of appropriate safety behaviors.	Intervention: Safety Culture Change Model Aims: “To improve pediatric patient safety at a tertiary, 200-bed children’s hospital by changing the safety culture and implementing processes, practices, and measures to sustain improvements.” [Descriptive]	**C:** Neither **M:** Yes **S:** No 1 hospital US 2 years	Internal authors’ assessment. **Interviews:** Staff members (included physicians and other clinical and administrative staff) (n = 100) **Focus Groups:** None reported **Surveys:** “Press Gainey [sic] Safety Culture Perception Survey” (n not reported); only scores for entire hospital system and not just this hospital were available* Other: Serious safety events (SSE),* VAP bundle,* Hand hygiene compliance,* Asthma core measures,* Medication errors*	**B:** None reported **E:** None reported **C:** Healthcare Performance Improvement Safety culture change model	Yes, based on reduced serious safety events and improved clinical process measures.	Capability Development, Communication, Culture, Facilitation/consultation, Goals, Leadership, Monitoring and evaluation, Multiple factors, Organization and structure, Organizational learning, Staff engagement, Sustainment
Roberts et al. (2020) A Public Health Performance Excellence Improvement Strategy: Diffusion and Adoption of the Baldrige Framework Within Tennessee Department of Health	Transformation not explicitly mentioned.	Intervention: Baldrige Performance Excellence Aims: “Report on the use of the Baldrige Performance Excellence Program as a framework to guide organizational change in public health departments” [Descriptive]	**C:** Neither **M:** No **S:** No 95 local health departments and unspecified number of county health departments US 7 years	Internal authors’ self-report **Interviews:** None reported **Focus Groups:** None reported **Surveys:** Association of State and Territorial Health Officials’ national employee workforce interests and needs survey in years 3 and 6 (n’s not reported)* Other: Review of progress reports to assess integration of performance improvement	**B:** Baldrige **E:** Baldrige **C:** Baldrige	Yes. “The transition from a previous compliance-based to the new improvement-based environment led to successful change that was both internally and externally recognized	Alignment, Capability Development, Community/ patient engagement, Culture, Impetus for change, Intentional change process, Leadership, Organizational learning, Staff engagement, Sustainment
Journal of Public Health Management Practice				principles into health departments’ programs,* Adoption measured through records of training and unit participation in rapid improvement events and completion of Baldrige applications,* Financial sustainability,* External recognition*		and linked to important population health improvements.”	
Shockey (2006) Norman Regional Hospital’s magnetic culture attracts employees, pleases customers, and keeps the business healthy Journal of Organizational Excellence	Transformation mentioned, but not explicitly defined. Refers to cultural transformation	Intervention: introduce a “magnetic culture” in a (struggling) regional hospital Aims: Aim of article not directly stated [Descriptive]	**C:** Comparison (of employee satisfaction survey scores to other Press-Ganey hospitals) **M:** Yes **S:** No 1 hospital US 5 years	Human Resources manager description of the transformation **Interviews:** None reported **Focus Groups:** Pre-intervention groups of 14 – 16 employees (n not reported) Groups of physicians (n not reported) **Surveys:** Annual Press Ganey Employee satisfaction surveys (Baseline n=638, post-intervention n=1589)* Physician satisfaction survey (final 2 years) Annual patient satisfaction survey **Other:** Employee retention and attraction metrics, financial performance	**B:** None reported **E:** None reported **C:** None reported	Yes. Improved employee satisfaction (“most direct measure of transformation”), staff retention and attraction, physician satisfaction, patient satisfaction	Communication, Culture, Leadership, Organization and structure, Staff engagement
Simons et al. (2015) Does lean management improve patient safety culture? An extensive evaluation of safety culture in a radiotherapy institute. European Journal of Oncology Nursing	Transformation not explicitly mentioned. Refers to change in patient safety culture.	Intervention: Lean management, Manchester Patient Safety Framework (MaPSaF) Aims: Determine if lean management improves patient safety culture in a radiotherapy institute. [Descriptive]	**C:** Neither **M:** Yes **S:** No 1 radiotherapy institute Netherlands 3 years	Internal and external authors’ assessment **Interviews:** Staff from different professions (n = 10)* Focus groups: 5 patient safety professionals (n = 1) **Surveys:** 3 waves of data collection using Hospital Survey on Patient Safety Culture (HSOPSC) (n = 54, 53, 51 respectively)* and Patient safety awareness and behavior survey (Developed in this study) (n = 52, 43, 30 respectively)*	**B:** Cooper’s Reciprocal safety culture model, Patient safety culture **E:** MaPSaF **C:** Lean, MaPSaF	Yes. “Patient safety culture improved significantly due to undertaken lean activities and the reorganization to managing care pathways . . .”	Alignment, Culture, Intentional change process, Leadership, Organization and structure, Staff engagement
				**Other:** Incident reporting system reports,* Workshops to assess patient safety culture*			
Stetler et al. (2009) Institutionalizing evidence-based practice: an organizational case study using a model of strategic change. Implementation Science	Transformation mentioned, but not explicitly defined. Refers to “EBP [Evidence-based practice] institutionalization . . .” as an example for transformation.	Intervention: Implement EBP Aims: “. . . this theoretically-based study sought to identify key contextual elements and related configurations and relationships in an organization where EBP was perceived to be used routinely, in contrast to one in which it was not” [Analytic]	**C:** Comparison **M:** No **S:** Yes 2 hospitals US 5 years	External authors’ purposeful selection of 2 sites to provide contrast, using input from a panel of experts from American Organization of Nurse Executives (AONE) and self-rated institutionalization scale measuring integration of EBP into structures and routines* Interviews: Leaders and individuals in 2 sites (n = 113) **Focus groups:** Staff from each site (n = 14 groups, 41 individuals) **Surveys:** Nursing staff and manager surveys—organizational learning survey (OLS), Multi-dimensional leader questionnaire (MLQ), Nursing work index/practice environment survey (PES), Research utilization (RU) (n = 143 at “role model site” and n = 86 at “beginner site”) **Other:** Observations of groups, document review, organizational performance measures	**B:** None reported **E:** Pettigrew et al. Strategic change **C:** None reported	Varied. Sites selected based on their extent of transformation – 1 high and 1 low.	Culture, Intentional change process, Leadership, Multiple factors, Staff engagement
Timmons et al. (2019) Provider strategies on ten elements of organizational transformation. Journal of Vocational Rehabilitation	Transformation mentioned but not explicitly defined. Refers to “organizational structures and service delivery models from primarily sheltered work to community-based work” and “. . . moving to integrated community services necessitates a complete rethinking of mission, vision, values, and practices.” (Citing Rogan and Rinne)	Intervention: Training and technical assistance for providers to transform from shelter-based work to community-based work Aims: Determine what implementation strategies each organization used in its transformation and what strategies they would recommend to others [Descriptive]	**C:** Neither **M:** No **S:** Yes 4 community mental health centers US 10 years, timeframe not completely clear	External authors’ study of sites selected by panel of experts based on previous success. **Interviews:** individual and group interviews (n = 41) with leadership, middle management, direct support or frontline staff, individuals with Intellectual and developmental disabilities, family members, and external stakeholders **Focus Groups:** Included with report of interviews and not distinguished from interviews **Surveys:** None reported	**B:** Training and Technical Assistance for Providers (T-TAP), Lyons et al. Expanded T-TAP model **E:** None reported **C:** None reported	Yes. The four sites were selected by external panel of experts based on their previous success in transitioning from shelter-based work to community-based work for clients	Capability Development, Communication, Community/patient engagement, Culture, Goals, Leadership, Staff engagement
				**Other:** Expert consensus on ten elements of successful transformation from Delphi panel and their nomination of sites*			
Todic et al. (2022) Critical Theory, Culture Change, and Achieving Health Equity in Health Care Settings Academic Medicine	Transformation mentioned, but not explicitly defined. Focus on transforming to “culture of equity”	Intervention: Advancing health equity Aims: “. . . describe 5 interconnected change strategies . . . [used] to build a culture of equity” [Descriptive]	**C:** Neither **M:** Yes **S:** No 1 academic medical center—hospital and medical school US 10 years	Internal authors’ self-report **Interviews:** Key informant interviews (n = 40)* Focus Groups: None reported **Surveys:** Diversity and equity survey with committee members from across the organization (n = 40)* Middle management survey (n = 105)* Annual employee diversity engagement survey (n not reported)* Other: Quality metrics stratified by race, ethnicity, zip code, gender, language and payer status,* Measurement of objective DEI goals [i.e., workforce is representative of the community]*	**B:** intersectionality, relational-cultural theory, and critical consciousness **E:** Health equity implementation framework **C:** Organizational Strategies for Building Cultures of Equity—Model specific to this study	Yes. ”We have been using the DEI theory of change . . . for 10 years, have accomplished multiple process outcomes, and are beginning to see outcomes consistent with our DEI strategic goals.”	Alignment, Capability Development, Community/patient engagement, Culture, Intentional change process
Venturato et al. (2019) Development and evaluation of an organisational culture change intervention in residential aged care facilities. Australasian Journal on Ageing	Transformation not explicitly mentioned. Refers to change in workplace culture: “. . . to develop more effective workplace cultures that have embedded within them person-centred processes, systems and ways of working.” (cites McCormack et al.)	Intervention: Towards Organisational Culture Change (TOrCCh) Aims: “. . . address the need for sustainable culture change . . . by developing and piloting a novel workforce development intervention . . . that would empower staff to identify and address the need for improved teamwork, communication and leadership . . .” [Descriptive]	**C:** Neither **M:** No **S:** No 5 long-term care facilities Australia Length of time for transformation not reported	Evaluation by external authors based on interviews and focus groups. **Interviews:** Facility managers (n = 6)* Focus groups: Staff (n = 20 groups)* Surveys: None reported **Other:** Observations and informal conversations with staff*	**B:** McCormack et al. Practice development **E:** Action research **C:** TOrCCh—model specific to this study. Action research	Yes. “. . . The project was perceived to have achieved its goal of impacting positively upon organizational culture . . .”	Capability Development, Communication, Culture, Intentional change process, Leadership, Staff engagement
Wolf (2011) Constructing rapid transformation: sustaining high performance and a new view of organization change. International Journal of Training & Development	Definition based on Morgan (1997): “Transformational change ultimately involves the creation of ‘new contexts’ that can break the hold of the dominant attractors in favor of new ones.”	Intervention: “Organic” transformation process Aims: Determine what supports the sustainability of high performance [Analytic]	**C:** Comparisons **M:** Yes, not reported **S:** Yes. High performing sites selected based on system’s performance measures. 12 hospitals; 9 sustaining change, 3 not US 5 years	External evaluation. Sustaining and non-sustaining facilities identified from corporate performance measures over time. **Interviews:** at 12 facilities with CEO, staff and directors or managers with long tenure (n = 41). **Focus Groups:** None reported **Surveys:** None reported **Other:** Corporate measures of employee engagement, turnover, patient satisfaction,* productivity,* outcomes including quality (no additional specification)* and financial performance*	**B:** Positive Discourse, Positive organizational change based on the core themes of Appreciative Inquiry, Wolf’s Baseline study of these 12 facilities **E:** None reported **C:** Model developed in this study based on appreciative inquiry	Varied. 9 sustaining and 3 non-sustaining identified from corporate performance measures prior to collection of qualitative data	Culture, Impetus for change, Intentional change process, Leadership, Multiple factors, Organizational learning, Staff engagement, Sustainment
Wong et al. (2002) Providing the right infrastructure to lead the culture change for patient safety. Joint Commission Journal on Quality Improvement	Transformation not explicitly mentioned. Refers to culture change.	Intervention: Promote a culture of safety and implement a safety program. Aims: Illustrate the program’s infrastructure and identify key factors for a successful patient safety program. [Descriptive]	**C:** Neither **M:** Yes **S:** No 1 hospital US 1 year	Internal authors’ report on their experience leading the change. **Interviews:** None reported **Focus groups:** None reported **Surveys:** Monthly surveys of members of hospital safety board (n not reported)* Other: Not reported although there is reference to patient safety measures	**B:** None reported **E:** None reported **C:** None reported	Yes. Improvement in self-assessment safety board survey measures.	Alignment, Culture, Intentional change process, Leadership, Organization and structure, Staff engagement

### RQ1: General Characteristics of the Literature on Organizational Transformation

The articles varied substantially in both scope and study aims. Examples are reducing restraints in a mental health setting, leveling power differentials between clinical professionals and their clients and community, improving diversity, equity and inclusion, and increasing patient safety. Based on their aims, we classified 14 (39%) articles as analytic and 22 (61%) as descriptive. The oldest article was published in 1996. The publication rate increased from zero to two articles annually through 2012, to one to three annually from 2013 to 2022. Most articles published after 2012 were descriptive (13/17, 76%). Twenty articles (56%) were published after the Best et al. (2012) and Lee et al. (2013) reviews. Only three of the included articles were also included in the prior Best et al. (2012), Lee et al. (2013), or Harrison et al. (2021) reviews on organizational transformation. Lukas et al. (2007) was included in all three, Henry et al. (2017) was included in Harrison et al. (2021), and McNulty and Ferlie (2004) was included in Lee et al. (2013).

Most articles (21, 58%) were set in hospitals. No other setting had more than four (11%) articles. Most articles were from the United States (20, 56%), the United Kingdom (7, 19%), and Canada (5, 14%). Articles were published in a wide range of journals. Most were the sole publication included from their respective journal. Exceptions were three articles in the *Joint Commission Journal on Quality & Patient Safety* and two in *Academic Medicine*. The highest number of articles (9, 25%) were in clinical journals (e.g., *American Journal of Community Psychology*), followed by eight (22%) in health care management (e.g., *Australian Health Review*) (see Supplementary File 2 for classification of journals). The numbers of sites ranged from one to 95. Most (23, 64%) were single-site studies. Three (8%) articles had more than 12 sites. Length of time for transformation ranged from 0.5 to 15 years, with a median of 3.5 and a mode of 3 years. The largest percentage (42%) of articles reported transformation lengths between 2 and 3.5 years, although four (11%) reported less than 2 years and eight (22%) over 7 years. Equal numbers (16, 44%) of articles had exclusively external authors and exclusively internal; four (11%) had both internal and external authors. Five studies were designed as qualitative comparison studies. They had different levels of success among sites (“varied” success in Table 1) and gave in-depth analysis of transformation processes in individual sites (Charns et al., 2022; Hunter et al., 2014; Lukas et al., 2007; Stetler et al., 2009; Wolf, 2011).

#### Analytic Versus Descriptive Aims

Analytic and descriptive articles differed with respect to authorship, number of sites, journal, research design, use of models, methods, measures, and success of transformation. They appear to represent different subsets of the transformation literature. Of the analytic articles, only one of 14 (7%) had exclusively internal authors and less than half (6/14, 43%) were single site. In contrast, most descriptive articles (15/22, 68%) had only internal authors and were single site (17/22, 77%). Studies with the largest number of sites (over 12) were in three descriptive articles, and the four other large studies (7 to 12 sites) were analytic. Journals primarily publishing analytic articles were in the fields of organization sciences (5/6, 83%) and health services research (3/4, 75%) and the journal *Implementation Science* (1/1); those primarily publishing descriptive articles were in the fields of health care quality and safety (6/6), health care management (7/8, 88%), and clinical disciplines (6/9, 67%).

The stated aims of analytic articles addressed understanding the dynamics of transformation or of factors affecting transformation, relationships between the intervention and contextual factors, people’s lived experience of creating change, or factors affecting sustainability of change. In contrast, aims of most descriptive articles were to present accounts of what was done. Aims of a few articles were to evaluate a particular program or intervention, and those of another few articles were clinically focused (e.g., “achieve baby friendly hospital designation” (Henry et al., 2017) without a stated research aim.

### RQ2: How Has Organizational Transformation Been Conceptualized and Defined?

Articles varied in their conceptualization and definition of transformation. Only four articles included explicit definitions of transformation (Charns et al., 2022; Lukas et al., 2007; McNulty & Ferlie, 2004; Wolf, 2011). Most (24, 67%) referred to transformation and included only general descriptions of transformation. These definitions and descriptions shared a focus on holistic change, emphasizing significant and pervasive shifts in how the organization functions. Most descriptions highlighted “culture change” involving values, behaviors, and beliefs that reflect a fundamental shift in the way people think and work. In addition, they highlighted the impact on ideologies, structures, processes, and power dynamics, signifying deep and fundamental change. Many descriptions further underscored the importance of system-wide strategic orientation, implying that transformation is a deliberate process aimed at achieving long-term improvements across whole organizations. While continuous improvement is explicitly mentioned in the context of Lean thinking, it is an underlying theme in many articles, portraying transformation as an ongoing and dynamic process.

Articles explicitly mentioning transformation (24, 67%) tended to focus directly on large-scale, holistic changes in organizational culture, values, and principles. In contrast, articles not explicitly mentioning transformation (12, 33%) tended to focus on organization-wide but more narrowly focused improvements to achieve specific health outcomes (e.g., reducing restraints, achieving baby-friendly hospital designation) without framing these changes within the context of a broader transformation. Nearly all analytic (13/14, 93%) and half of descriptive articles (11/22) explicitly mention transformation.

### RQ3: How Has Organizational Transformation Been Studied and Measured?

#### Study Design

Few articles (9, 25%) used either comparison or control sites. Most (22, 61%) measured transformation before and after the transformation process. Very few articles (5, 14%) reported using measures for site selection. Nine (25%) reported having none of these three research design attributes.

#### Methods and Measures of Transformation

There were 12 qualitative, 10 quantitative, and 14 mixed-methods studies. Half of all articles (18) reported using staff/employee surveys. Following in frequency were interviews/focus groups of staff/employees (14, 39%), interviews/focus groups of managers (8, 22%), observations (7, 19%), document review (7, 19%), quality/safety measures (7, 19%), and surveys/interviews of patients/consumers (6, 17%). Four articles (11%) used external assessments of transformation (e.g., independent panel of experts). Articles reported a range of measures related to organizational transformation. Some used direct measures of transformation, such as changes in staff engagement and in organizational culture. Others focused on indirect or downstream outcomes presumed to reflect successful transformation, including clinical or quality performance indicators, patient satisfaction, financial metrics, and external recognition (e.g., accreditation or awards). Strictly speaking, the clinical process and outcome measures and other organizational performance metrics were not measures of transformation *per se*, but rather were indicators of factors expected to be affected by transformation. We report them here only to reflect this literature. At most, three studies used similar surveys, an NHS staff survey; however, lack of detail in the articles precludes a definitive conclusion that they used the identical survey. Two each referred to Press Ganey surveys and AHRQ safety culture surveys; again, it is not clear if these references are to the same surveys. Some articles used measures unique to that article and setting, such as research performance, awards, human resource metrics, and achievement of trust status. One study (Charns et al., 2022) developed a composite measure of transformation based on 11 “markers” of transformation (e.g., staff engagement, staff empowerment, culture of respect) assessed for both their “depth” or “rigor of application” and “spread throughout the organization.”

#### Models Used for Evaluation

Nineteen articles (53%) used models for evaluation. Of these 17 (47%) reported using one or more published models. Only four published models were mentioned in more than one article: Action Research (4), Pettigrew et al.’s Strategy and Change (3), Complex Organizational Change (2), and Diffusion of Innovation (2) (Details in Supplement 2). Ten (28%) articles mentioned 11 published models that were not mentioned in any other article. Four (11%) used models for evaluation developed within that study. Use of models for evaluation increased over time; most articles (7/9, 78%) in the latest period (2018–2022) used some model for evaluation.

#### Analytic vs Descriptive Aims

Analytic studies were more rigorous, used different methods and measures, and provided richer descriptions of the transformation process than descriptive studies. A higher percentage of analytic (5/14, 36%) than descriptive articles (4/22, 18%) used comparisons or controls. Analytic studies primarily used qualitative methods (9/14, 64%) and interviews/focus groups with staff (9/14, 64%). Only one analytic study (1/14, 7%) used quality/safety measures and two (2/14, 14%) used staff surveys. In contrast, descriptive studies primarily used quantitative (9/22, 41%) or mixed methods (10/22, 45%), quality/safety measures (6/22, 27%), and staff surveys (16/22, 73%). The analytic articles using comparisons provided in-depth reports of differences in transformation processes. In contrast, the descriptive articles using comparison or control sites—including the three large multi-site studies noted in RQ1 (Benning et al., 2011; Jones et al., 2013; Roberts et al., 2020)—focused on staff survey results at intervention sites compared to quantitative data aggregated from multiple comparison sites and provided minimal information on the transformation process in comparison sites.

There was not a direct correspondence between the rigor of research design and articles’ contributions to understanding of transformation. Of the nine articles that had none of the three Higgins et al.’s attributes—and therefore the weakest research design—five were analytic (Bess et al., 2009; Brooks, 1996; McNulty & Ferlie, 2004; Ochocka et al., 1999; Peirson et al., 2012) and four descriptive (Corring et al., 2016; Lord et al., 1998; Roberts et al., 2020; Venturato et al., 2019). These represented 36% of all analytic articles (5 of 14) and 18% of all descriptive articles (4 of 22). All five of the analytic articles used qualitative methods and provided rich descriptions of transformation. Seven of the nine articles, including all five of the analytic articles, were evaluations that included authors external to the organizations. These external authors did not select the sites for study, engage other sites for comparison or measure before the transformation process. Six of the nine articles used qualitative (67%), two mixed (22%), and one quantitative methods (11%). That only one quantitative article had none of the Higgins et al.’s attributes is because all 10 quantitative articles included measurement of changes in survey scores and only one of these (Roberts et al., 2020) did not measure before the start of the transformation process.

A much greater proportion of analytic (11/14, 79%) than descriptive articles (8/22, 36%) used a model for evaluation. All three mentions of Pettigrew et al.’s strategy and change and two mentions of complex organizational change as evaluation models were in analytic articles.

Use of models for evaluation was more prevalent in articles with external authors and in health services research and clinical journals, and lower proportion of articles reporting single-site studies (see Supplemental File 2 for all detailed breakdowns of methods and measures).

### RQ4: What Are Dynamics and Factors Important to Organizational Transformation?

#### Analysis of Conclusions About Transformation

In the directed qualitative content analysis, we coded 492 text segments extracted from the articles’ conclusions and qualitative results. We applied codes from 20 of the 21 constructs of the OTM and created 12 inductive codes, resulting in 32 unique codes. In each article, we coded between one and 45 segments, with an average of 14 per article. Most prevalent codes across articles were “Leadership support and commitment” (28 articles, 78%), “Organization culture” (22, 61%), “Frontline staff engagement and enthusiasm for transformation” (21, 58%), “Alignment of organizational strategy, policies and resources” (17, 47%), “Interaction of multiple factors” (16, 44%), “Capability development” (16, 44%), and “Middle manager engagement and enthusiasm for transformation” (14, 39%). Six of these seven codes were coded deductively from the OTM; “Interaction of multiple factors” was an inductive code. Other inductively identified codes were mentioned in few articles (see Table 2 for exemplary quotes).

**Table 2.** Conclusions About Transformation.

Thematic category Code	Exemplary quote	N (%)
**Alignment**		**17 (47%)**
Alignment of organizational strategy, policies and resources	“The inclusion, in the strategic planning process, of lean **goals that, subsequently, have been translated into measurable objectives comprised in the annual budget planning.**” (Centauri et al., 2018)	17 (47%)
Improvement efforts are aligned with organizational strategy	“Over time, **performance improvement approaches were integrated into existing departmental programs and new initiatives**, leading to additional successful process changes and population health improvements.” (Roberts et al., 2020)	1 (3%)
**Capability Development**		**16 (44%)**
Capability development	“Concerted **effort to develop necessary skills** and enabling systems and resources before expecting any group to be effective in participatory decision making . . .” (Best et al., 1997)	16 (44%)
**Communication**		**11 (31%)**
Communication regarding the transformation	“Communication has three areas of importance. It is a **repeated reinforcement of the centrality** of Lean in the medical center’s work; it amplifies the True North goals, **setting the context for improvement** activities; and it **informs staff** of Lean initiatives’ accomplishments to convey that real change is possible.” (Charns et al., 2022)	11 (31%)
**Community/patient engagement**		**11 (31%)**
Community/patient engagement in Transformation activities	“The inclusiveness of the process **involving family, consumers, board members, staff members and the community as equal decision making partners** at the table solidified the commitment to the process and to the plan that was created.” (Lord et al., 1998)	11 (31%)
**Culture**		**24 (67%)**
Culture of respect; just culture	“The synergistic effects of our safety culture-change initiative also led to new levels of involvement, accountability, and transparency at both the leadership and at the unit levels. Staff at all levels gained a new understanding of the behaviors essential to preventing harm to our patients.” (Peterson et al., 2012)	9 (25%)
Organization culture	“The project demonstrates a design to deliver compassionate person- and family-centred care through multifactorial microsystems cultural transformation . . . It illustrates service culture change at the frontline, where the interface between staff and patients directly affects delivery of safe and effective, person-centred care.” (McKellar et al., 2020)	22 (61%)
**Facilitation/consultation**		**6 (17%)**
Facilitation/consultation	“**Skilled facilitation was initially provided by external consultants** who were able to engage staff in a co-creation process. The coaching model increasingly transitioned facilitation to embedded staff, supporting sustainability.” (McKellar et al., 2020)	6 (17%)
**Goals**		**11 (31%)**
Values	“Collectively held values underpin organizational culture. It is not surprising that attention to the organizational value system became a top priority for the management team.” (Brooks 1996)	9 (25%)
Vision	“Using their new **vision as the core of their messaging**, these providers recognized the importance of transparency and the reality that they needed to reassure stakeholders who were resistant. Through **consistent and clear explanation of their new organizational goals**, they could engage a broad range of stakeholders both internally (individuals, families, front-line staff) and externally (employers, community collaborators) in a way that created momentum and built a coalition of invested partners.” (Timmons et al., 2019)	5 (14%)
**Impetus for change**		**10 (28%)**
Abduction	“. . .Discovery, which entails moving away from prevailing habits and moving toward new ones, operates via five abduction sequences. **Individuals in turn can sustain the discovery process via constructively oriented responses to surprise and to resulting discoveries** (Golden-Biddle, 2020)	2 (6%)
Impetus to transform	“Thus, the failed merger may have created a **‘burning platform’ i.e., a collective sense of urgency** that actually facilitated the acceptance of sweeping change.” (Kirch et al., 2006)	9 (25%)
**Intentional change process**		**14 (39%)**
Adaptation	“Continual evolution and adaptability are critical to success in culture change efforts. **Flexibility to deviate from the planned agenda** to identify and apply what worked supported success.” (McKellar et al., 2020)	2 (6%)
Rigorous use of Transformation tools and techniques	“Outcomes from the process and systems focus (e.g., **use of PDCAs, strategy maps, Lean**, etc) become organizational lessons learned that are reapplied through knowledge management shared across diverse departmental units to underscore expectations of improvements-based results.” (Roberts et al., 2020)	5 (14%)
Use of change model	[Negative] “Even though there was a broadly shared understanding of the programme’s theory of change (what the intervention was intended to achieve and with what methods), **there was less evidence of an explicit and shared organisational theory of change** (what was needed to make the programme work at an organisational level and how programme implementation could be optimised).” (Benning et al., 2011)	10 (28%)
**Leadership**		**29 (81%)**
Change champions	“As the programme continues to gain momentum, there is a sense that **Quality and Safety Champions** are part of something and hence a social movement is developing.” (Foster, 2016)	6 (17%)
Leadership support and commitment to Lean	“An **interdisciplinary leadership team and the board of trustees** are critical stakeholders and must play an integral role in defining and approving the principles and behavioral standards.” (Connor et al., 2007)	28 (78%)
Shared leadership	“The **leadership of change was thus something that was shared across the organization**, as the critical mass of support for change began to grow and develop, and as natural local line leaders began to emerge from a number of different points.” (Bate et al., 2000)	4 (11%)
**Monitoring and evaluation**		**9 (25%)**
Availability of data/metrics	“Staff can see, through the **use of measurement**, that they are making a difference.” (Foster, 2016)	7 (19%)
Informed decision making	“[Transformational change] demands a **knowledge management strategy to ensure evidence and decision making information is current, comprehensive, accessible, usable and evaluable**.” (Peirson et al., 2012)	4 (11%)
**Multiple factors**		**16 (44%)**
Interaction of multiple factors	“The analysis showed that **several organizational and external factors played a relevant role in letting the change program be implemented. But no specific factor was sufficient** by itself to explain the outcomes of the lean change programs.” (Centauri et al., 2018)	16 (44%)
**Organization and structure**		**13 (36%)**
Integration across intraorganizational boundaries	“The application of lean methodology in contexts where there is an ongoing process to overcome the vertical traditional organization with the **introduction of integrated and multidisciplinary clinical pathways** and the adoption of more patients-centered healthcare delivery models . . .” (Centauri et al., 2018)	8 (22%)
Interdepartmental collaboration	[Negative] “There is much less evidence of interconnected and coherent process redesign across the hospital as a healthcare system than espoused in the [program] rhetoric. [. . .] it remained the case that **process management and ongoing attention to process redesign occurred within the already established framework** of clinical specialties and clinical directorates.” (McNulty & Ferlie, 2004)	4 (11%)
Structure	“Culture and structure should be co-produced. Culture is central throughout the process. **Structure holds changes in place rather than creates change**.” (Bate et al., 2000)	9 (25%)
**Organizational learning**		**11 (31%)**
Learning organization and priority on continuous learning	“[This transformation approach] emphasizes the importance of **continuous and collective learning** and experimentation . . . ” (Bate et al., 2000)	11 (31%)
**Staff engagement**		**26 (72%)**
Cultivating positive sentiments	“. . . Homan’s (1951/1992) group development cycle of a required **activity, which leads to an interaction, resulting in a sentiment, which impacts our further perspectives** on that action in the future.” (Wolf, 2011)	3 (8%)
Frontline staff empowerment to make decisions to improve process	“Organisations should seek to **build capacity of staff to resolve issues themselves.**” (Foster, 2016)	9 (25%)
Frontline staff engagement and enthusiasm for Transformation	[Negative] **“Few of the frontline ward staff members interviewed, however, seemed aware of PDSA cycles.** Somewhere between the blunt end and the sharp end, the model of participative engagement on which SPI1 was based had got rather lost, **at least in relation to medical wards.”** (Benning et al., 2011)	21 (58%)
Improvement Initiatives (projects, Transformation process improvement)	“Departmental units documented **performance improvement through hundreds of internal projects** and more than 100 innovation-driven Baldrige achievement awards.” (Roberts et al., 2020)	7 (19%)
Middle manager engagement and enthusiasm for Transformation	“The stable guidance, sponsorship and support, at all organizational levels, with the top management launching and sustaining lean as a strategic pillar, and **first-line managers influencing other colleagues to be involved.**” (Centauri et al., 2018)	14 (39%)
**Sustainment**		**9 (25%)**
Sustainment	[Negative] “Although modest gains were achieved, t**his demonstration project failed to result in sustainable culture change** because insufficient attention was paid to Kotter’s step 7 and 8 of organisational change [consolidating improvements and producing still more change, institutionalizing new approaches]. This would have required better project planning and closer integration between the Health Hospital project and overall hospital operations.” (Best et al., 1997)	9 (25%)
**Time**		**6 (17%)**
Change takes time	“The primary lesson learned is that this type of **fundamental attitude change of mental healthcare providers takes time, patience and determination**.” (Corring et al., 2016)	6 (17%)

We organized codes into 17 thematic categories to produce a hierarchical coding structure (see Table 2). Categories in the largest number of articles were “Leadership” (29, 81%), “Staff engagement” (26, 72%) and “Culture” (24, 67%). Categories most frequently employed conjointly (i.e., appearing in the same article) were “Leadership” and “Staff Engagement” (23 articles, 64%) (e.g., “leadership walk rounds were intended as opportunities to connect meaningfully with staff, but were perceived as failing to achieve meaningful connection” (Benning et al., 2011, negative example)), “Leadership” and “Culture” (21, 58%) (e.g., “a focus on distributed leadership underpinned with an appreciative inquiry approach was used to promote organizational values” (Kerr et al., 2015)), and “Culture” and “Staff Engagement” (19, 53%) (e.g., “a ‘bottom-up’ approach based on principles of distributed leadership, promoting staff engagement, and encouraging continuous learning contributed to sustained culture change” (McKellar et al., 2020)). Codes from these three categories—“Leadership,” “Staff engagement,” and “Culture”—appeared conjointly in half of the articles. The coded segments revealed thematic relations among the three categories. “**Leadership**” segments highlighted the significance of distributed, shared, and collaborative leadership. Inclusive and participatory approaches were cited as crucial to engage, give a voice and a sense of ownership to employees at all levels through strategies like steering committees, communication channels, and “change champions.” “**Staff engagement**” segments mentioned the crucial role of staff collaboration in project planning and ensuring that frontline expertise guides new processes, thereby promoting staff ownership, buy-in, and commitment to the change process. The use of staff projects was mentioned as a specific staff engagement strategy. The key role of middle managers in change processes was also highlighted, underscoring the importance of capacity building and engagement of this specific group. “**Culture**” segments underscored the importance of prioritizing and proactively promoting positive culture through multifaceted approaches including symbolic leadership actions, communicating shared values and purpose, and staff engagement at all levels to overcome initial resistance to change. Together, segments from these three categories suggest that a balance of top-down strategic direction coupled with bottom-up, participatory approaches are required to engage staff in meaningful ways to support culture change and achieve organizational transformation.

#### Models Used to Guide Change

Twenty-three articles (64%) used models to guide change. Twenty articles (56%) used a previously published model. The most mentioned model used for change (8, 22%) was a Quality Improvement (QI) model, including Lean, Virginia Mason Production Systems (VMPS, that incorporates Lean); and the Institute for Healthcare Improvement (IHI) change model. Four (11%) used Action Research and an additional article used Transactive Planning, built on Action Research. Seven (19%) articles mentioned a published model not mentioned in any other article (Appreciative Inquiry, Baldrige, HPI, Integrated Knowledge Translation, Kotter, Process Reengineering, TeamSTEPPS) (see Supplementary File 2 for detail and citations to models).

A greater proportion of analytic (12/14, 86%) than descriptive (11/22, 50%) articles used a model for change. The use of models for change increased over time, with 17 of 24 articles (71%) published after 2007 using a model for change, compared to six of 12 (50%) from 1996 to 2007. All (7) studies having seven or more sites used a model, whereas only half of single-site studies (12/23, 52%) used a model for change. Models were used to guide change in a higher proportion of articles with external authors, and in those published in health services research, organization science, and clinical journals.

### RQ5: To What Extent Have Transformation Efforts in Health Care as Represented in the Literature Been Successful?

Most articles (23/36, 64%) reported successful transformation, two partial (6%), and six unsuccessful (17%) transformation. Five (14%) studies were multi-site studies designed to compare sites having different levels of success (i.e., “varied success”). After removing these five studies from the calculation, 74% (23/31) of articles reported full success.

The reasons for failure of transformation were diverse. Examples include challenges “achieving full ‘buy-in’ . . . from all staff” (Barba et al., 2021), inability of both staff and community members to overcome traditional power differences in two community-based health and human services organizations (Bess et al., 2009), inability to sustain gains in participatory decision making for staff over 3 years in a hospital (Best et al., 1997), and failure of process reengineering in a hospital, attributed to a prior structural change that had the unintended consequence of incentivizing middle managers not to support the change (McNulty & Ferlie, 2004).

When comparing reported success against different article characteristics, we found differences in reported success by aims (analytic vs. descriptive), authorship, journal, setting, country, use of a model for evaluation, methods, research design, and measures. We did not find differences based on number of sites or use of a model to guide change.

Descriptive articles reported a higher proportion of success (17/22, 77%) and either success or partial success (19/22, 86%) than analytic (6/9, 67%) articles (No analytic articles reported partial success). Articles with exclusively internal authors reported a higher proportion of success (13/16, 81%) and either success or partial success (15/16, 94%) than those with exclusively external authors (8/11, 73%) or both types of authors (2/4, 50%). All articles published in academic medicine (2) and health care quality/safety (6) journals reported successful transformation. A high proportion of articles in clinical journals also reported success (7/9, 78%), as did articles in organization science journals (4/5, 80%). The greatest variation in reported success was in articles in health care management journals (3/7, 43% successful, 2/7, 29% partially successful, 2/7, 29% unsuccessful). Only health services research (2), health care management (1), implementation science (1) and organization science (1) journals published comparative studies with varied success. All studies in academic medical centers (2), specialty institutes (2), and health care systems (1) were successful. Success rates in other settings ranged from 50% to 75%, but small numbers of studies in each setting preclude meaningful comparisons. Small numbers of studies in some countries also preclude meaningful comparisons; however, we note differences among the United States (14/16, 88% successful), the United Kingdom (4/6 67% successful), and Canada (2/5, 40% successful and 1 partially successful, yielding 3/5, 60% either successful or partially successful). Articles not using a model for evaluation (13/16, 81%) reported more success than those that did (10/15, 67%). The highest proportion of success was reported in studies using quantitative methods (9/10, 90%), compared with those using qualitative methods (7/10, 70%) or mixed methods (7/11, 64% successful, 9/11, 82% successful or partially successful). All three studies selecting sites based on measures and two using comparison sites reported success; this contrasts with studies having none of the three features of research design in which five (56%) reported success and six (67%) reported success or partial success. All studies measuring success with external assessment (3), quality/safety measures (6), organizational performance (1), or financial performance (2) reported success. The proportion of studies reporting success was high for those using staff surveys (14/18, 78% successful, 15/18, 83% successful or partially successful). The proportion of success in studies using various qualitative measures ranged from 64% to 80%.

## Discussion

### Summary of Main Findings

This systematic review identified 36 articles on organizational transformation in health care. A very small percentage of all initially identified articles met our inclusion criteria, primarily because they were not organization-wide or did not provide information on the transformation process, which was an important aim of this review. We also note that for these reasons there was minimal overlap between the articles included in this review and earlier reviews by Best et al. (2012), Lee et al. (2013), and Garritsen et al. (2024).

### RQ1: General Characteristics of the Literature on Organizational Transformation

There has been a very modest increase in the number of publications per year starting in 2012. This suggests that despite the growing interest in organizational transformation in health care, it is still under-researched. Although studies were conducted in six countries, most were from the United States, the United Kingdom, and Canada. Consistent with previous reviews, most studies were in hospitals, highlighting a gap in knowledge about transformation in other health care settings.

The literature on transformation is notably fragmented, characterized by the lack of concentration of articles in any particular journal. Furthermore, most studies were single-site, and the foci of transformation varied greatly. Many articles deviated from conventional scientific formats, presenting narrative reflections by senior leaders of the change efforts themselves. While these articles offer valuable insights into leadership perspectives, they may also be biased in their accounts and lack the rigor required for in-depth evaluation of the transformation process and outcomes. In addition, the reported duration of transformation initiatives varied widely, with 25% of the articles reporting periods as short as 2 years or less—a period we consider unrealistic given the complexity of large-scale organizational change. Such studies may either underestimate the success of transformational efforts that are evaluated prematurely before achieving their full potential or be overly optimistic in assessing success or in estimating time required for actual whole organization transformation.

Articles fell into two distinct subsets: analytic and descriptive. This distinction was reflected in the types of journals that publish them and their research methods and measures. Analytic articles applied overall more rigorous designs, and used qualitative methods, whereas descriptive articles were typically weaker in design, relied on quantitative surveys or performance metrics, and were more frequently authored by internal leaders.

### RQ2: How Has Organizational Transformation Been Conceptualized and Defined?

Organizational transformation, although not always explicitly labeled as such, was frequently described as a fundamental cultural change or shift in organizational functioning. Only four of 36 articles included definitions of transformation, similar to Garritsen et al.’s (2024) findings. Instead, transformation was often framed within the specific context of each study, without a clear or standardized definition. Also, we found transformation was widely discussed but lacked conceptual clarity, and consistent application of frameworks was limited; this underscores the need for a more cohesive and systematic approach to studying health care transformation.

### RQ3: How Has Organizational Transformation Been Studied and Measured?

Many included articles lacked detail in describing their methods and measures, and there was heterogeneity in methods and measures across articles. Studies often had weak research designs. Very few common measures were used across studies. While several studies used surveys to assess culture and staff engagement, most survey measures were unique to their context and not comparable. Similarly, a few studies used quality/safety measures, also unique to the studies. Lack of consistency in measurement makes it challenging to compare results across studies. In addition, measures of quality/safety and of organizational performance are not direct measures of transformation itself. Instead, they are measures that are assumed to be affected by transformation. Ideally, articles would include measures of factors expected to affect transformation (e.g., leadership), measures of transformation (e.g., culture change), and measures of organizational outcomes (e.g., improved patient safety).

To improve the robustness of research on organizational transformation, future studies should consider adopting stronger methodological designs, including longitudinal and mixed-methods approaches that include measurement of factors such as organizational culture and employee engagement before and after (and at times during) the transformation, that can capture the complexity and temporal dynamics of change processes. In addition, using comparison or control sites and embedding realist or process evaluation components can offer insights into how and why transformation efforts succeed or fail in particular contexts. We also suggest that the Higgins et al.’s criterion for use of measures for site selection be broadened to “assessment of sites for site selection” to include methods other than quantitative ones, such as use of expert panels. The use of conceptual models (e.g., Organizational Transformation Model) can help clarify mechanisms and guide both data collection and analysis. For example, studies such as Lukas et al. (2007) and Charns et al. (2022) provide useful examples by combining conceptual models with empirical rigor in the evaluation of large-scale transformation initiatives. Use of conceptual models also provides a vehicle for different studies to build upon each other in reporting empirical findings and for testing relationships comprising the complex dynamics of transformation. Descriptive studies that provide rich description of model constructs and their interrelationships can make important contributions to understanding transformation, much more so than descriptive studies that do not use conceptual models or theories.

### RQ4: What Are Dynamics and Factors Important to Organizational Transformation?

Articles’ conclusions about transformation noted “Leadership,” “Staff engagement,” “Culture,” and combinations thereof as central to transformation. Noteworthy is the intertwining of leadership and staff engagement, specifically the focus on distributed leadership across the hierarchy and efforts to enable frontline staff to fully engage with and own transformation efforts. Changing culture is central to most definitions of organizational transformation and is consistent with Pettigrew’s findings that core beliefs are pivotal to how a firm functions and that strategic change, therefore, involves changing these core beliefs (Sminia, 2016). Harrison et al. (2022) also discuss “affective commitment” of staff, that is, “a want or desire to support the change recognising the benefits associated with it.” This conceptually is an extension of “staff engagement.” These three constructs are key elements of the OTM and are consistent with the first of the “five simple rules” from the Best et al. (2012) review: (1) “engage individuals at all levels in the change efforts,” (2) “establish feedback loops,” (3) “attend to history,” (4) “engage physicians,” and (5) “involve patients and families.” Physician engagement was included in several articles but was not explicitly mentioned in articles’ conclusions that we coded. The other “simple rules” were infrequently mentioned.

Our findings differ from the three prior reviews in that few of the included articles mentioned either context or history, whereas Best et al. (2012) specifically included context and history, Lee et al. (2013) included antecedents, and Garritsen et al. (2024) included conditions for transformation. Articles included in our review that did include history or context made clear their effects on transformation, either as a factor that contributed to failure of transformation or as a stimulus, which we coded as “Impetus to transform.”

The inconsistent use of conceptual models to guide and evaluate organizational transformation is an important characterization of this literature. Not only were few models mentioned in more than one article, but also several models that were mentioned are not models of transformation (e.g., IHI QI model, TeamSTEPPS). Based on this review, no particular conceptual model has been shown to be a better representation of the realities of transformation than any other. Future research should address development and testing of models of transformation.

Using the OTM as a framework for coding articles’ conclusions allowed a broad approach to identify factors affecting transformation, as the OTM has a large number of constructs, and we inductively added additional codes. Our findings were largely consistent with the constructs of the OTM, with 19 of 21 OTM constructs coded in more than one article. OTM constructs specific to Lean transformation appeared rarely (e.g., “rigorous use of tools and techniques,” generalized from “rigorous use of Lean tools and techniques”), or never at all (i.e., “use of standard work to increase reliability and decrease variation”) despite our effort to generalize these constructs beyond the Lean context. Beyond the factors of leadership, staff engagement, and culture, we did not find as frequent mention of other factors describing the transformation process. One reason for this is that—despite our inclusion criterion of a description of the transformation process—several articles did not address this well, focusing instead on documenting outcomes without a clear articulation of how transformation unfolded.

### RQ5: To What Extent Have Transformation Efforts in Health Care as Represented in the Literature Been Successful?

Most included articles reported positive outcomes of transformation, suggesting that publication bias as represented in this literature is likely. Exceptions were five multi-site comparative studies, designed to compare transformation processes. Successful transformation was more commonly reported in articles that were descriptive, had internal authors, were published in academic medicine, health care quality and safety, clinical and organization science journals, did not use a model for evaluation, and used quantitative methods and measures. Many of these articles concluded transformation was successful based on performance measures rather than direct measures of transformation.

### Strengths and Limitations

A limitation of this study is that despite a comprehensive search strategy, it is possible that not all relevant articles were identified. For example, the search did not include books that may have reported on transformation in depth. The primary literature itself has several weaknesses: the lack of detail in many of the included articles, inconsistent structure of articles making it difficult to extract information, and likely a bias toward successful transformation. Despite these limitations, this review also has notable strengths. Our search strategy encompassed both medical and business databases, ensuring a broad scope of the literature. To enhance reliability of our findings, two reviewers conducted every phase of data collection and analysis, discussing discrepancies and referring back to the original sources. We also examined several factors that might be related to bias. In addition, we achieved a more accurate description of studies’ designs by reporting their design features rather than relying on study design labels that are often applied inconsistently in the literature.

## Conclusion

This review highlights the pressing need for more rigorous study and detailed description of efforts to transform health care organizations. Despite recognition of the importance of organizational transformation in health care, the literature is fragmented. Gaps identified in prior reviews a decade ago remain. Articles often lacked cohesive frameworks, consistent measures, and rigorous analyses. We strongly advocate that future studies test and build upon published models of transformation. Models provide a structure for accumulation of empirical findings across research studies. We also suggest that future studies include direct measures, such as employee engagement, of the transformation process, as well as rich descriptions of the interaction of factors affecting transformation. The absence of model-building and consistent measurement in this field may be a consequence of the dispersed nature of this literature, spanning a wide range of journals and disciplines. Lack of consistency in use of models and measures are impediments to advancement of research on organizational transformation.

The future of health care depends on our ability to innovate and transform at the organizational level. Rather than investing tremendous resources in the implementation of individual evidence-based practices and isolated quality improvements, organizational transformation efforts are needed to fundamentally shift the culture and way health care organizations function—thereby collectively facilitating introduction of new practices.

## Supplemental Material

sj-docx-1-mcr-10.1177_10775587251356130 – Supplemental material for Defining and Measuring Organizational Transformation in Health Care: A Systematic Literature Review Supplemental material, sj-docx-1-mcr-10.1177_10775587251356130 for Defining and Measuring Organizational Transformation in Health Care: A Systematic Literature Review by Lauren Clack, Jason Smith and Martin Charns in Medical Care Research and Review

sj-docx-2-mcr-10.1177_10775587251356130 – Supplemental material for Defining and Measuring Organizational Transformation in Health Care: A Systematic Literature Review Supplemental material, sj-docx-2-mcr-10.1177_10775587251356130 for Defining and Measuring Organizational Transformation in Health Care: A Systematic Literature Review by Lauren Clack, Jason Smith and Martin Charns in Medical Care Research and Review

sj-docx-3-mcr-10.1177_10775587251356130 – Supplemental material for Defining and Measuring Organizational Transformation in Health Care: A Systematic Literature Review Supplemental material, sj-docx-3-mcr-10.1177_10775587251356130 for Defining and Measuring Organizational Transformation in Health Care: A Systematic Literature Review by Lauren Clack, Jason Smith and Martin Charns in Medical Care Research and Review

sj-docx-4-mcr-10.1177_10775587251356130 – Supplemental material for Defining and Measuring Organizational Transformation in Health Care: A Systematic Literature Review Supplemental material, sj-docx-4-mcr-10.1177_10775587251356130 for Defining and Measuring Organizational Transformation in Health Care: A Systematic Literature Review by Lauren Clack, Jason Smith and Martin Charns in Medical Care Research and Review
