# Supplementary material for: Defining and Measuring Organizational Transformation in Health Care: A Systematic Literature Review
Source: Med Care Res Rev. 2025 Aug 13;83(2):71–102. doi: 10.1177/10775587251356130 (PMC12946223; doi:10.1177/10775587251356130)
Supplement: sj-docx-2-mcr-10.1177_10775587251356130 – Supplemental material for Defining and Measuring Organizational Transformation in Health Care: A Systematic Literature Review [file sj-docx-2-mcr-10.1177_10775587251356130.docx]

**Table A: Classification of Journals**

| **Type of Journal** | **Name of Journal** | **Number of Articles** |
| --- | --- | --- |
| Academic Medicine | *Academic Medicine* | 2 |
| Health Services Research | *British Medical Journal (BMJ)*  *Health Services and Delivery Research*  *Health Services Management Research*  *Health Services Research* | 1  1  1  1 |
| Health Care Management | *Australian Health Review*  *Future Hospital Journal*  *Healthcare Management Forum*  *Health Care Management Review*  *Healthcare Quarterly*  *International Journal of Healthcare Management*  *Journal of Public Health Management Practice*  *Management in Health Care* | 1  1  1  1  1  1  1  1 |
| Health Care Quality & Safety | *BMJ Quality & Safety*  *Joint Commission Journal on Quality & Patient Safety*  *Joint Commission Journal on Quality Improvement (Renamed Joint Commission Journal on Quality & Patient Safety)*  *Journal of Healthcare Risk Management*  *Journal of Patient Safety* | 1  2  1  1  1 |
| Implementation Science | *Implementation Science* | 1 |
| Organization Science | *Academy of Management Journal*  *International Journal of Training & Development*  *Journal of Organizational Excellence*  *Leadership & Organization Development Journal*  *Organization Science*  *Organization Studies* | 1  1  1  1  1  1 |
| Clinical | *American Journal of Community Psychology*  *Australasian Journal on Ageing*  *BMC Public Health*  *Canadian Journal of Community Mental Health*  *European Journal of Oncology Nursing*  *Psychiatric Rehabilitation Journal*  *Journal of Vocational Rehabilitation*  *Journal of Child and Adolescent Psychiatric Nursing*  *Nursing for Women’s Health* | 1  1  1  1  1  1  1  1  1 |

**Table B: Article Characteristics and Crosstabs by Aims and Models**

| **Article Characteristics** | **N**  **---**  **% of total for column** | **Aims**  **N**  **% of row**  **% of column** | | **Use Model for Evaluation** | | **Use Model to Guide Change** | |
| --- | --- | --- | --- | --- | --- | --- | --- |
|  |  | Analytic | Descriptive | Yes | No | Yes | No |
| **Total Articles** | 36 | 14  39%  --- | 22  61%  --- | 19  53%  --- | 17  47%  --- | 23  64%  --- | 13  36  --- |
| **Period of Publication** |  |  |  |  |  |  |  |
| 1996-2002 | 6  17% | 3  50% (3/6)*  21% (3/14)** | 3  50% (3/6)*  14% (3/22)*** | 4  67%  21% | 2  33%  12% | 4  67%  17% | 2  33%  15% |
| 2003-2007 | 6  17% | 3  50%  21% | 3  50%  14% | 2  33%  11% | 4  67%  24% | 2  33%  9% | 4  67%  31% |
| 2008-2012 | 7  19% | 4  57%  29% | 3  43%  14% | 3  43%  16% | 4  57%  24% | 5  71%  22% | 2  29%  15% |
| 2013-2017 | 8  22% | 1  13%  7% | 7  88%  32% | 3  38%  16% | 5  63%  29% | 6  75%  26% | 2  25%  15% |
| 2018-2022 | 9  25% | 3  33%  21% | 6  67%  27% | 7  78%  37% | 2  22%  12% | 6  67%  26% | 3  33%  23% |
|  |  |  |  |  |  |  |  |
| **Setting** |  |  |  |  |  |  |  |
| Academic medical center | 2  6% | 1  50%  7% | 1  50%  5% | 1  50%  5% | 1  50%  6% | 1  50%  4% | 1  50%  8% |
| Community health | 4  11% | 2  50%  14% | 2  50%  9% | 3  75%  16% | 1  25%  6% | 3  75%  13% | 1  25%  8% |
| Healthcare system | 3  8% | 3  100%  21% | 0 | 3  100%  16% | 0 | 3  100%  13% | 0 |
| Hospital | 21  58% | 7  33%  50% | 14  67%  64% | 7  33%  37% | 14  67%  82% | 12  57%  52% | 9  43%  69% |
| Long-term care | 2  6% | 0 | 2  100%  9% | 2  100%  11% | 0 | 1  50%  4% | 1  50%  8% |
| Public health | 2  6% | 1  50%  7% | 1  50%  5% | 2  100%  11% | 0 | 2  100%  9% | 0 |
| Specialty institute | 2  6% | 0 | 2  100%  9% | 1  50%  5% | 1  50%  6% | 1  50%  4% | 1  50%  8% |
|  |  |  |  |  |  |  |  |
| **Country** |  |  |  |  |  |  |  |
| Australia | 2  6% | 0 | 2  100%  9% | 2  100%  11% | 0 | 1  50%  4% | 1  50%  8% |
| Canada | 5  14% | 2  40%  14% | 3  60%  14% | 4  80%  21% | 1  20%  6% | 3  60%  13% | 2  40%  15% |
| Italy | 1  3% | 1  100%  7% | 0 | 1  100%  5% | 0 | 1  100%  4% | 0 |
| Netherlands | 1  3% | 0 | 1  100%  5% | 1  100%  5% | 0 | 1  100%  4% | 0 |
| UK | 7  19% | 4  57%  29% | **3**  43%  14% | 3  43%  16% | 4  57%  24% | 7  100%  30% | 0 |
| US | 20  56% | 7  35%  50% | 13  65%  59% | 8  40%  42% | 12  60%  71% | 10  50%  43% | 10  50%  77% |
|  |  |  |  |  |  |  |  |
| **Type of journal** |  |  |  |  |  |  |  |
| Academic medicine | 2  6% | 1  50%  7% | 1  50%  5% | 1  50%  5% | 1  50%  6% | 1  50%  4% | 1  50%  8% |
| Health services research | 4  11% | 3  75%  21% | 1  25%  5% | 3  75%  16% | 1  25%  6% | 4  100%  17% | 0 |
| Healthcare management | 8  22% | 1  13%  7% | 7  88%  32% | 4  50%  21% | 4  50%  24% | 4  50%  17% | 4  50%  31% |
| Healthcare quality & safety | 6  17% | 0 | 6  100%  27% | 1  17%  5% | 5  82%  29% | 2  33%  9% | 4  67%  31% |
| Implementation science | 1  3% | 1  100%  7% | 0 | 1  100%  5% | 0 | 0 | 1  100%  8% |
| Organization science | 6  17% | 5  83%  36% | 1  17%  5% | 3  50%  16% | 3  50%  18% | 5  83%  22% | 1  17%  8% |
| Clinical | 9  25% | 3  33%  21% | 6  67%  27% | 6  67%  32% | 3  33%  18% | 7  78%  30% | 2  22%  15% |
|  |  |  |  |  |  |  |  |
| **Number of sites** |  |  |  |  |  |  |  |
| 1 | 23  64% | 6  26%  43% | 17  74%  77% | 9  39%  47% | 14  61%  82% | 12  52%  52% | 11  48%  85% |
| 2-5 | 6  17% | 4  67%  29% | 2  33%  9% | 5  83%  26% | 1  17%  6% | 4  67%  17% | 2  33%  15% |
| 7-12 | 4  11% | 4  100%  29% | 0 | 3  75%  16% | 1  25%  6% | 4  100%  17% | 0 |
| >12 | 3  8% | 0 | 3  100%  14% | 2  67%  11% | 1  33%  6% | 3  100%  13% | 0 |
|  |  |  |  |  |  |  |  |
| **Length of time for transformation (years)** |  |  |  |  |  |  |  |
| <2 | 4  11% | 2  50%  14% | 2  50%  9% | 3  75%  16% | 1  25%  6% | 3  75%  13% | 1  25%  8% |
| 2-3.5 | 15  42% | 6  40%  43% | 9  60%  41% | 6  40%  32% | 9  60%  53% | 11  73%  48% | 4  27%  31% |
| 4-7 | 8  22% | 3  38%  21% | 5  63%  23% | 3  38%  16% | 5  63%  29% | 2  25%  9% | 6  75%  46% |
| >7 | 8  22% | 3  38%  21% | 5  63%  23% | 6  75%  32% | 2  25%  12% | 6  75%  26% | 2  25%  15% |
| Not Reported | 1  3% | 0 | 1  100%  5% | 1  100%  5% | 0 | 1  100%  4% | 0 |
|  |  |  |  |  |  |  |  |
| **Author** |  |  |  |  |  |  |  |
| External | 16  44% | 12  75%  86% | 4  25%  18% | 12  75%  63% | 4  25%  24% | 14  88%  61% | 2  13%  15% |
| Internal | 16  44% | 1  6%  7% | 15  94%  68% | 3  19%  16% | 13  81%  76% | 6  38%  26% | 10  63%  77% |
| Both internal & external | 4  11% | 1  25%  7% | 3  75%  14% | 4  100%  21% | 0 | 3  75%  13% | 1  25%  8% |
|  |  |  |  |  |  |  |  |
| **Aims** |  |  |  |  |  |  |  |
| Analytic | 14  39% | X | X | 11  79%  58% | 3  21%  18% | 12  86%  52% | 2  14%  15% |
| Descriptive | 22  61% | X | X | 8  36%  42% | 14  64%  82% | 11  50%  48% | 11  50%  85% |
|  |  |  |  |  |  |  |  |
| **Mention Transformation** |  |  |  |  |  |  |  |
| Yes | 24  67% | 13  54%  93% | 11  46%  50% | 14  58%  74% | 10  42%  59% | 16  67%  70% | 8  33%  62% |
| No | 12  33% | 1  8%  7% | 11  92%  50% | 5  42%  26% | 7  58%  41% | 7  58%  30% | 5  42%  38% |

Key: * Value in cell (3) divided by row total (6)

** Value in cell (3) divided by column total (14)

*** Value in cell (6) divided by column total (22)

**Table C: Crosstabs of Aims, Research Design, Methods, Measures & Models**

| **Article Characteristics** | **N**  **% of total for column** | **Aims**  **N**  **% of row**  **% of column** | | **Research Design** | | | | | | | **Methods** | | | **Use Model for Evaluation** | | **Use Model to Guide Change** | |
| --- | --- | --- | --- | --- | --- | --- | --- | --- | --- | --- | --- | --- | --- | --- | --- | --- | --- |
|  |  |  | | **Use Comparison or control sites** | | **Measure before & after trans.** | | **Select sites based on measures** | | **Has None of 3 Aspects** |  |  |  |  |  |  |  |
|  |  | **Analytic** | **Descriptive** | **Yes** | **No** | **Yes** | **No** | **Yes** | **No** |  | **Qual** | **Quant** | **Mixed** | **Yes** | **No** | **Yes** | **No** |
| **Total Articles** | 36 | 14  39% | 22  61% | 9  25% | 27  75% | 22  61% | 14  39% | 5  14% | 31  86% | 9  25% | 12  33% | 10  28% | 14  39% | 19  53% | 17  47% | 23  64% | 13  36% |
|  |  |  |  |  |  |  |  |  |  |  |  |  |  |  |  |  |  |
| **Research design** |  |  |  |  |  |  |  |  |  |  |  |  |  |  |  |  |  |
| Comparison sites | 7  19% | 5  71%  36% | 2  29%  9% | 7  100%  78% | 0 | 5  71%  23% | 2  29%  14% | 2  29%  40% | 5  71%  16% |  | 2  29%  17% | 1  14%  10% | 4  57%  29% | 4  57%  21% | 3  43%  18% | 5  71%  22% | 2  29%  15% |
| Control sites | 2  6% | 0 | 2  100%  9% | 2  100%  22% | 0 | 2  100%  9% | 0 | 0 | 2  100%  6% |  | 0 | 1  50%  10% | 1  50%  7% | 1  50%  5% | 1  50%  6% | 2  100%  9% | 0 |
| Comparison or control sites | 9  25% | 5  56%  36% | 4  44%  18% | X | X | 7  78%  32% | 2  22%  14% | 2  22%  40% | 7  78%  23% | 0 | 2  22%  17% | 2  22%  20% | 5  56%  36% | 5  56%  26% | 4  44%  24% | 7  78%  30% | 2  22%  15% |
| Neither comparison nor control | 27  75% | 9  33%  64% | 18  67%  82% | X | X | 15  56%  68% | 12  44%  86% | 3  11%  60% | 24  89%  77% | 9  33%  100% | 10  37%  83% | 8  30%  80% | 9  33%  64% | 14  52%  74% | 13  48%  76% | 16  59%  70% | 11  41%  85% |
| Measure before & after | 22  61% | 5  23%  36% | 17  77%  77% | 7  32%  78% | 15  68%  56% | X | X | 1  5%  20% | 21  95%  68% | 0 | 4  18%  33% | 9  41%  90% | 9  41%  64% | 8  36%  42% | 14  64%  82% | 12  55%  52% | 10  45%  77% |
| Do not measure before & after | 14  39% | 9  64%  64% | 5  36%  23% | 2  14%  22% | 12  86%  44% | X | X | 4  29%  80% | 10  71%  32% | 9  64%  100% | 8  57%  67% | 1  7%  10% | 5  36%  36% | 11  79%  58% | 3  21%  18% | 11  79%  48% | 3  21%  23% |
| Select sites based on measures | 5  14% | 4  80%  29% | 1  20%  5% | 2  40%  22% | 3  60%  11% | 1  20%  5% | 4  80%  29% | X | X | 0 | 2  40%  17% | 0 | 3  60%  21% | 3  60%  16% | 2  40%  12% | 3  60%  13% | 2  40%  15% |
| Do not select sites based on measures  (incl 1 NR) | 31  86% | 10  32%  71% | 21  68%  95% | 7  23%  78% | 24  77%  89% | 21  68%  95% | 10  32%  71% | X | X | 9  29%  100% | 10  32%  83% | 10  32%  100% | 11  35%  79% | 16  52%  84% | 15  28%  88% | 20  65%  87% | 11  35%  85% |
| None | 9  25% | 5  56%  36% | 4  44%  18% | 0 | 9  100%  33% | 0 | 9  100%  64% | 0 | 9  100%  29% | X | 6  67%  50% | 1  11%  10% | 2  22%  14% | 7  78%  37% | 2  22%  12% | 8  89%  35% | 1  11%  8% |
|  |  |  |  |  |  |  |  |  |  |  |  |  |  |  |  |  |  |
| **Methods** |  |  |  |  |  |  |  |  |  |  |  |  |  |  |  |  |  |
| Qualitative | 12  33% | 9  75%  64% | 3  25%  14% | 2  17%  22% | 10  83%  37% | 4  33%  18% | 8  67%  57% | 2  17%  40% | 10  83%  32% | 6  50%  67% | X | X | X | 9  75%  47% | 3  25%  18% | 11  92%  48% | 1  8%  8% |
| Quantitative | 10  28% | 1  10%  7% | 9  90%  41% | 2  20%  22% | 8  80%  30% | 9  90%  41% | 1  10%  7% | 0 | 10  100%  32% | 1  10%  11% | X | X | X | 2  20%  11% | 8  80%  47% | 4  40%  17% | 6  60%  46% |
| Mixed | 14  39% | 4  29%  29% | 10  71%  45% | 5  36%  56% | 9  64%  33% | 9  64%  41% | 5  36%  36% | 3  21%  60% | 11  79%  35% | 2  14%  22% | X | X | X | 8  57%  42% | 6  43%  35% | 8  57%  35% | 6  43%  46% |
|  |  |  |  |  |  |  |  |  |  |  |  |  |  |  |  |  |  |
| **Measures of Transformation** |  |  |  |  |  |  |  |  |  |  |  |  |  |  |  |  |  |
| Interviews/ focus groups with staff | 14  39% | 9  64%  64% | 5  36%  23% | 3  21%  33% | 11  79%  41% | 6  43%  27% | 8  57%  57% | 1  7%  20% | 13  93%  42% | 6  43%  67% | 9  64%  75% | 0 | 5  36%  36% | 14  100%  74% | 0 | 13  93%  57% | 1  7%  8% |
| Interviews/focus groups with managers | 8  22% | 7  88%  50% | 1  13%  5% | 3  38%  33% | 5  63%  19% | 3  38%  14% | 5  63%  36% | 1  13%  20% | 7  88%  23% | 3  38%  33% | 7  88%  58% | 0 | 1  13%  7% | 8  100%  42% | 0 | 8  100%  35% | 0 |
| Observations | 7  19% | 6  86%  43% | 1  14%  5% | 0 | 7  100%  26% | 1  14%  5% | 6  86%  43% | 1  14%  20% | 6  86%  19% | 5  71%  56% | 7  100%  58% | 0 | 0 | 6  86%  32% | 1  14%  6% | 7  100%  30% | 0 |
| Document review | 7  19% | 5  71%  36% | 2  29%  9% | 1  14%  11% | 6  86%  22% | 1  14%  5% | 6  86%  43% | 2  29%  40% | 5  71%  16% | 4  57%  44% | 4  57%  33% | 1  14%  10% | 2  29%  14% | 7  100%  37% | 0 | 7  100%  30% | 0 |
| External assessment | 4  11% | 1  25%  7% | 3  75%  14% | 1  25%  11% | 3  75%  11% | 1  25%  5% | 3  75%  21% | 2  50%  40% | 2  50%  6% | 1  25%  11% | 2  50%  17% | 0 | 2  50%  14% | 2  50%  11% | 2  50%  12% | 2  50%  9% | 2  50%  15% |
| Staff surveys | 18  50% | 2  11%  14% | 16  89%  73% | 4  22%  44% | 14  78%  52% | 15  83%  68% | 3  17%  21% | 1  11%  40% | 16  89%  51% | 2  11%  22% | 0 | 8  44%  80% | 10  56%  71% | 8  44%  42% | 10  56%  59% | 10  56%  43% | 8  44%  62% |
| Quality/safety measures | 7  19% | 1  14%  7% | 6  86%  27% | 1  14%  11% | 6  86%  22% | 7  100%  32% | 0 | 1  14%  20% | 6  86%  19% | 0 | 0 | 2  29%  20% | 5  71%  36% | 2  29%  11% | 5  71%  29% | 4  57%  17% | 3  43%  23% |
| Patient/consumer surveys/ interviews | 6  17% | 4  67%  29% | 2  33%  9% | 1  17%  11% | 5  83%  19% | 2  33%  9% | 4  67%  29% | 1  17%  20% | 5  83%  16% | 4  67%  44% | 2  33%  17% | 1  17%  10% | 3  50%  21% | 3  50%  16% | 3  50%  18% | 4  67%  17% | 2  33%  17% |
| Organizational performance | 2  6% | 2  100%  14% | 0 | 1  50%  11% | 1  50%  4% | 2  100%  9% | 0 | 1  50%  20% | 1  50%  3% | 0 | 0 | 1  50%  10% | 1  50%  7% | 0 | 2  100%  12% | 1  50%  4% | 1  50%  8% |
| Financial performance | 3  8% | 1  33%  7% | 2  67%  9% | 1  33%  11% | 2  67%  7% | 2  67%  9% | 1  33%  7% | 1  33%  20% | 2  67%  6% | 1  33%  11% | 0 | 2  67%  20% | 1  33%  7% | 1  33%  5% | 2  67%  12% | 2  67%  9% | 1  33%  8% |
|  |  |  |  |  |  |  |  |  |  |  |  |  |  |  |  |  |  |

| **Use Model for Evaluation** |  |  |  |  |  |  |  |  |  |  |  |  |  |  |  |  |  |
| --- | --- | --- | --- | --- | --- | --- | --- | --- | --- | --- | --- | --- | --- | --- | --- | --- | --- |
| Yes | 19  53% | 11  58%  79% | 8  42%  36% | 5  26%  56% | 14  74%  52% | 8  42%  36% | 11  58%  79% | 3  16%  60% | 16  84%  52% | 7  37%  78% | 9  47%  75% | 2  11%  20% | 8  42%  57% | X | X | 16  84%  70% | 3  16%  23% |
| Articles using published model* | 17  47% | 10  59%  71% | 7  41%  32% | 5  29%  56% | 12  71%  44% | 7  41%  32% | 10  59%  71% | 2  12%  40% | 15  88%  48% | 7  41%  78% | 9  53%  75% | 2  12%  20% | 6  35%  43% | X | X | 15  88%  65% | 2  12%  15% |
| Articles using published model not used in any other article | 10  28% | 4  40%  29% | 6  60%  27% | 3  30%  33% | 7  70%  26% | 6  60%  27% | 4  40%  29% | 1  10%  20% | 9  90%  29% | 3  30%  33% | 4  40%  33% | 2  20%  20% | 4  40%  29% | X | X | 9  90%  39% | 1  10%  8% |
| Action research | 4  11% | 3  75%  21% | 1  25%  5% | 0 | 4  100%  15% | 1  25%  5% | 3  75%  21% | 0 | 4  100%  13% | 3  75%  33% | 4  100%  33% | 0 | 0 | X | X | 4  100%  17% | 0 |
| Complex organizational change | 2  6% | 2  100%  14% | 0 | 1  50%  11% | 1  50%  4% | 1  50%  5% | 1  50%  7% | 0 | 2  100%  6% | 1  50%  11% | 2  100%  17% | 0 | 0 | X | X | 2  100%  9% | 0 |
| Strategy & change | 3  8% | 3  100%  21% | 0 | 2  67%  22% | 1  33%  4% | 0 | 3  100%  21% | 1  33%  20% | 2  67%  6% | 1  33%  11% | 1  33%  8% | 0 | 2  67%  14% | X | X | 2  67%  9% | 1  33%  8% |
| Diffusion of innovation | 2  6% | 1  50%  7% | 1  50%  5% | 2  100%  22% | 0 | 2  100%  9% | 0 | 0 | 2  100%  6% | 0 | 1  50%  8% | 1  50%  10% | 0 | X | X | 2  100%  9% | 0 |
| None | 17  47% | 3  18%  21% | 14  82%  64% | 4  24%  47% | 13  76%  48% | 14  82%  64% | 3  18%  21% | 2  12%  40% | 15  88%  48% | 2  12%  22% | 3  18%  35% | 8  47%  80% | 6  35%  43% | X | X | 7  41%  30% | 10  59%  77% |
|  |  |  |  |  |  |  |  |  |  |  |  |  |  |  |  |  |  |
| **Use Model to Guide Change** |  |  |  |  |  |  |  |  |  |  |  |  |  |  |  |  |  |
| Yes | 23  64% | 12  52%  86% | 11  48%  50% | 7  30%  78% | 16  70%  59% | 12  52%  55% | 11  48%  79% | 3  13%  60% | 20  87%  65% | 8  35%  89% | 11  48%  92% | 4  17%  40% | 8  35%  57% | 16  70%  84% | 7  30%  41% | X | X |
| Published model** | 20  56% | 10  50%  71% | 10  50%  45% | 6  30%  67% | 14  70%  52% | 10  50%  45% | 10  50%  71% | 2  10%  40% | 18  90%  58% | 7  35%  78% | 10  50%  83% | 4  20%  40% | 6  30%  43% | 15  75%  79% | 5  25%  29% | X | X |
| Published model not used in any other article | 8  22% | 2  25%  14% | 6  75%  27% | 2  25%  22% | 6  75%  22% | 5  63%  23% | 3  38%  21% | 0 | 8  100%  26% | 3  38%  33% | 3  38%  25% | 3  38%  30% | 2  25%  14% | 5  63%  26% | 3  38%  18% | X | X |
| Action Research | 5  14% | 3  60%  21% | 2  40%  9% | 0 | 5  100%  19% | 1  20%  5% | 4  80%  29% | 0 | 5  100%  16% | 4  80%  44% | 4  80%  33% | 0 | 1  20%  7% | 5  100%  26% | 0 | X | X |
| QI | 8  22% | 5  63%  36% | 3  38%  14% | 4  50%  44% | 4  50%  15% | 5  63%  23% | 3  38%  21% | 2  25%  40% | 6  75%  19% | 0 | 3  38%  25% | 1  13%  10% | 4  50%  29% | 6  75%  32% | 2  25%  12% | X | X |
| None | 13  36% | 2  15%  14% | 11  85%  50% | 2  15%  22% | 11  85%  41% | 10  77%  45% | 3  23%  21% | 2  15%  40% | 11  85%  35% | 1  8%  11% | 1  8%  8% | 6  46%  60% | 6  46%  43% | 3  23%  16% | 10  77%  59% | X | X |

* Published models used for evaluation:

1. Abduction (Locke, 2011; Locke et al., 2008; Peirce & Eisele, 1976; Peirce et al., 1931-1938; Peirce et al., 1992; Peirce et al., 1934; Peirce et al., 1935; Van Maanen et al., 2007)

2. Action research N=4 (Burnes, 1992; Coenen, 1988; French & Bell, 1984; Nelson et al., 1998; Perkins, 1995; Rappaport, 1981; Saegert & Winkel, 1996; Shani & Pasmore, 1985; Waterman et al., 2001; Yeich & Levine, 1992; Zimmerman, 2000; Zimmerman & Rappaport, 1988)

3. Baldrige Performance Excellence Program (Baldrige National Quality, 2005; US Department of Commerce, 2017)

4. Complex organizational change N=2 (Greenhalgh et al., 2004; Greenwood & Hinings, 1996; Grol et al., 2007; Poole & Van de Ven, 2004)

5. Deliberately developmental organization (Kegan & Lahey, 2016)

6. Diffusion of innovation N=2 (Rogers, 1995, 2003)

7. Evaluating Training Programs (Kirkpatrick & Kirkpatrick, 2006)

8. Health equity implementation framework (Woodward et al., 2019)

9. Manchester Patient Safety Framework (MaPSaF) (National Health Service National Patient Safety Agency, 2006)

10. Microsystem effectiveness (Donaldson & Mohr, 2001; Nelson et al., 2002)

11. Organization Transformation Model (OTM) (Azevedo et al., 2021; Lukas et al., 2007)

12. Psychological safety (Edmondson, 2018)

13. Roles consistent with shift in mental health programs to an empowerment focus (Carling, 1995)

14. Stages in the evidence-informed public health process (National Collaborating Centre for Methods and Tools, 2009)

15. Strategy and change N=3 (Ferlie et al., 1996; Iles & Sutherland, 2001; Pettigrew & Whipp, 1992; Pettigrew, 1985; Pettigrew, 1987; Pettigrew, 1992; Pettigrew et al., 1992)

**Table D: Crosstabs of Success of Transformation by Article Characteristics**

| **Article Characteristics** | **N**  **% of total for column** | **Success** | | | | **Total Articles without Varied Success** | **Success – Adjusted Percents Without Varied Success** | | |
| --- | --- | --- | --- | --- | --- | --- | --- | --- | --- |
|  |  | **Yes** | **Partial** | **No** | **Mixed** |  | **Yes** | **Partial** | **No** |
| **Total Articles** | 36 | 23  64% | 2  6% | 6  17% | 5  14% | 31 | 23  74% | 2  6% | 6  19% |
|  |  |  |  |  |  |  |  |  |  |
| **Aims** |  |  |  |  |  |  |  |  |  |
| Analytic | 14  39% | 6  43%  26% | 0 | 3  21%  50% | 5  36%  100% | 9  29% | 6  67%  26% | 0 | 3  33%  50% |
| Descriptive | 22  61% | 17  77%  74% | 2  9%  100% | 3  14%  50% | 0 | 22  71% | 17  77%  74% | 2  9%  100% | 3  14%  50% |
|  |  |  |  |  |  |  |  |  |  |
| **Authors** |  |  |  |  |  |  |  |  |  |
| Internal | 16  44% | 13  81%  57% | 2  13%  100% | 1  6%  17% | 0 | 16  52% | 13  81%  57% | 2  13%  100% | 1  6%  17% |
| External | 16  44% | 8  50%  35% | 0 | 3  19%  50% | 5  31%  100% | 11  35% | 8  73%  35% | 0 | 3  27%  50% |
| Both | 4  11% | 2  50%  9% | 0 | 2  50%  33% | 0 | 4  13% | 2  50%  9% | 0 | 2  50%  33% |
|  |  |  |  |  |  |  |  |  |  |
| **Journal** |  |  |  |  |  |  |  |  |  |
| Academic Medicine | 2  6% | 2  100%  9% | 0 | 0 | 0 | 2  6% | 2  100%  9% | 0 | 0 |
| Clinical | 9  25% | 7  78%  30% | 0 | 2  22%  33% | 0 | 9  29% | 7  78%  30% | 0 | 2  22%  33% |
| Health Services Research | 4  11% | 1  25%  4% | 0 | 1  25%  17% | 2  50%  40% | 2  6% | 1  50%  4% | 0 | 1  50%  17% |
| Health Care Management | 8  22% | 3  38%  13% | 2  25%  100% | 2  25%  33% | 1  13%  20% | 7  23% | 3  43%  13% | 2  29%  100% | 2  29%  33% |
| Health Care Quality / Safety | 6  17% | 6  100%  26% | 0 | 0 | 0 | 6  19% | 6  100%  26% | 0 | 0 |
| Implementation Science | 1  3% | 0 | 0 | 0 | 1  100%  20% | 0 | 0 | 0 | 0 |
| Organization Science | 6  17% | 4  67%  17% | 0 | 1  17%  17% | 1  17%  20% | 5  16% | 4  80%  17% | 0 | 1  20%  17% |
|  |  |  |  |  |  |  |  |  |  |
| **Setting** |  |  |  |  |  |  |  |  |  |
| Academic Medical Center | 2  6% | 2  100%  9% | 0 | 0 | 0 | 2  6% | 2  100%  9% | 0 | 0 |
| Community Health | 4  11% | 3  75%  13% | 0 | 1  25%  17% | 0 | 4  13% | 3  75%  13% | 0 | 1  25%  17% |
| Health Care System | 3  8% | 1  33%  4% | 0 | 0 | 2  67%  40% | 1  3% | 1  100%  4% | 0 | 0 |
| Hospital | 21  58% | 13  62%  57% | 1  5%  50% | 4  19%  67% | 3  14%  60% | 18  58% | 13  72%  57% | 1  6%  50% | 4  22%  67% |
| Long-term Care | 2  6% | 1  50%  4% | 1  50%  50% | 0 | 0 | 2  6% | 1  50%  4% | 1  50%  50% | 0 |
| Public Health | 2  6% | 1  50%  4% | 0 | 1  50%  17% | 0 | 2  6% | 1  50%  4% | 0 | 1  50%  17% |
| Specialty Institute | 2  6% | 2  100%  9% | 0 | 0 | 0 | 2  6% | 2  100%  9% | 0 | 0 |
|  |  |  |  |  |  |  |  |  |  |
| **Country** |  |  |  |  |  |  |  |  |  |
| Australia | 2  6% | 1  50%  4% | 1  50%  50% | 0 | 0 | 2  6% | 1  50%  4% | 1  50%  50% | 0 |
| Canada | 5  14% | 2  40%  9% | 1  20%  50% | 2  40%  33% | 0 | 5  16% | 2  40%  9% | 1  20%  50% | 2  40%  33% |
| Italy | 1  3% | 1  100%  4% | 0 | 0 | 0 | 1  3% | 1  100%  4% | 0 | 0 |
| The Netherlands | 1  3% | 1  100%  4% | 0 | 0 | 0 | 1  3% | 1  100%  4% | 0 | 0 |
| UK | 7  19% | 4  57%  17% | 0 | 2  29%  33% | 1  14%  20% | 6  19% | 4  67%  17% | 0 | 2  33%  33% |
| US | 20  56% | 14  70%  61% | 0 | 2  10%  33% | 4  20%  80% | 16  52% | 14  88%  61% | 0 | 2  13%  33% |
|  |  |  |  |  |  |  |  |  |  |
| **Number of Sites** |  |  |  |  |  |  |  |  |  |
| 1 | 23  64% | 17  74%  74% | 2  9%  100% | 4  17%  67% | 0 | 23  74% | 17  74%  74% | 2  9%  100% | 4  17%  67% |
| 2-5 | 6  17% | 4  67%  17% | 0 | 1  17%  17% | 1  17%  20% | 5  16% | 4  80%  17% | 0 | 1  20%  17% |
| 7-12 | 4  11% | 0 | 0 | 0 | 4  100%  80% | 0 | 0 | 0 | 0 |
| >12 | 3  8% | 2  67%  9% | 0 | 1  33%  17% | 0 | 3  10% | 2  67%  9% | 0 | 1  33%  17% |
|  |  |  |  |  |  |  |  |  |  |
| **Use Model for Evaluation** |  |  |  |  |  |  |  |  |  |
| Yes | 19  53% | 10  53%  43% | 1  5%  50% | 4  21%  67% | 4  21%  80% | 15  48% | 10  67%  43% | 1  7%  50% | 4  27%  67% |
| No | 17  47% | 13  76%  57% | 1  6%  50% | 2  12%  33% | 1  6%  20% | 16  52% | 13  81%  57% | 1  6%  50% | 2  13%  33% |
|  |  |  |  |  |  |  |  |  |  |
| **Use Model to Guide Change** |  |  |  |  |  |  |  |  |  |
| Yes | 23  64% | 15  65%  65% | 0 | 4  17%  67% | 4  17%  80% | 19  61% | 15  79%  65% | 0 | 4  21%  67% |
| No | 13  36% | 8  62%  35% | 2  15%  100% | 2  15%  33% | 1  8%  20% | 12  39% | 8  67%  35% | 2  17%  100% | 2  17%  33% |
|  |  |  |  |  |  |  |  |  |  |
| **Research Methods** |  |  |  |  |  |  |  |  |  |
| Qualitative | 12  33% | 7  58%  30% | 0 | 3  25%  50% | 2  17%  40% | 10  32% | 7  70%  30% | 0 | 3  30%  50% |
| Quantitative | 10  28% | 9  90%  39% | 0 | 1  10%  17% | 0 | 10  32% | 9  90%  39% | 0 | 1  10%  17% |
| Mixed Methods | 14  39% | 7  50%  30% | 2  14%  100% | 2  14%  33% | 3  21%  60% | 11  35% | 7  64%  30% | 2  18%  100% | 2  18%  33% |
|  |  |  |  |  |  |  |  |  |  |
| **Research Design** |  |  |  |  |  |  |  |  |  |
| Comparison sites | 7  19% | 2  29%  9% | 0 | 0 | 5  71%  100% | 2  6% | 2  100%  9% | 0 | 0 |
| Control sites | 2  6% | 1  50%  4% | 0 | 1  50%  17% | 0 | 2  6% | 1  50%  4% | 0 | 1  50%  17% |
| Comparison or control sites | 9  25% | 3  33%  13% | 0 | 1  11%  17% | 5  56%  100% | 4  13% | 3  75%  13% | 0 | 1  25%  17% |
| Measure before & after | 22  61% | 15  68%  65% | 1  5%  50% | 3  14%  50% | 3  14%  60% | 19  61% | 15  79%  65% | 1  5%  50% | 3  16%  50% |
| Select sites based on measures | 5  14% | 3  60%  13% | 0 | 0 | 2  40%  40% | 3  10% | 3  100%  13% | 0 | 0 |
| None | 9  25% | 5  56%  22% | 1  11%  50% | 3  33%  50% | 0 | 9  29% | 5  56%  22% | 1  11%  50% | 3  33%  50% |
|  |  |  |  |  |  |  |  |  |  |
| **Measures** |  |  |  |  |  |  |  |  |  |
| Interviews/ focus groups with staff | 14  39% | 7  50%  30% | 0 | 4  29%  67% | 3  21%  60% | 11  35% | 7  64%  30% | 0 | 4  36%  67% |
| Interviews/focus groups with managers | 8  22% | 4  50%  17% | 0 | 1  13%  17% | 3  38%  60% | 5  16% | 4  80%  17% | 0 | 1  20%  17% |
| Observations | 7  19% | 5  71%  22% | 0 | 2  29%  33% | 0 | 7  23% | 5  71%  22% | 0 | 2  29%  33% |
| Document review | 7  19% | 4  57%  17% | 0 | 2  29%  33% | 1  14%  20% | 6  19% | 4  67%  17% | 0 | 2  33%  33% |
| External assessment | 4  11% | 3  75%  13% | 0 | 0 | 1  25%  20% | 3  10% | 3  100%  13% | 0 | 0 |
| Staff surveys | 18  50% | 14  78%  61% | 1  6%  50% | 3  17%  50% | 0 | 18  58% | 14  78%  61% | 1  6%  50% | 3  17%  50% |
| Quality/safety measures | 7  19% | 6  86%  26% | 0 | 0 | 1  14%  20% | 6  19% | 6  100%  26% | 0 | 0 |
| Patient/Consumer Interviews/Surveys | 6  17% | 3  50%  13% | 1  17%  50% | 1  17%  17% | 1  17%  20% | 5  16% | 3  60%  13% | 1  20%  50% | 1  20%  17% |
| Organizational performance | 2  6% | 1  50%  4% | 0 | 0 | 1  50%  20% | 1  3% | 1  100%  4% | 0 | 0 |
| Financial performance | 3  8% | 2  67%  9% | 0 | 0 | 1  33%  20% | 2  6% | 2  100%  9% | 0 | 0 |

**References**

Agency for Healthcare Research and Quality. (2010). *TeamSTEPPS: strategies and tools to enhance performance and patient safety*. Retrieved 12 Jul 2010 from <http://www.teamstepps.ahrq.gov>

Azevedo, K. J., Gray, C. P., Gale, R. C., Urech, T. H., Ramirez, J. C., Wong, E. P., Lerner, B., Charns, M. P., & Vashi, A. A. (2021). Facilitators and barriers to the Lean Enterprise Transformation program at the Veterans Health Administration. *Health Care Manage Rev*, *46*(4), 308-318. <https://doi.org/10.1097/HMR.0000000000000270>

Baldrige National Quality, P. (2005). *Health care criteria for performance excellence*. Baldrige National Quality Program, NIST.

Bicen, P., & Johnson, W. H. (2015). Radical innovation with limited resources in high‐turbulent markets: The role of lean innovation capability. *Creativity and Innovation Management*, *24*(2), 278-299.

Burnes, B. (1992). *Managing change : a strategic-approach to organisational development and renewal*. Pitman London.

Bush, R. W. (2007). Reducing Waste in US Health Care Systems. *JAMA*, *297*(8), 871-874. <https://doi.org/10.1001/jama.297.8.871>

Bushe, G. (2012). Foundations of Appreciative Inquiry: History, Criticism and Potential. *AI Practitioner*, *14*(1).

Bushe, G. R. (2011). Appreciative inquiry: Theory and critique. In D. Boje, B. Burnes, & J. Hassard (Eds.), *The Routledge companion to organizational change* (pp. 87-103). Routledge.

Carling, P. J. (1995). *Return to community: Building support systems for people with psychiatric disabilities*. Guilford Press.

Coenen, H. (1988). On the foundations of a relationship based on equality between the researcher and the research party. In B. Boog, H. Coenen, L. Keune, & R. Lammerts (Eds.), *The complexity of relationships in action research* (pp. 17-35). Tillburg University Press.

Donaldson, M. S., & Mohr, J. J. (2001). *Exploring Innovation and Quality Improvement in Health Care Micro-Systems: A Cross-Case Analysis*. <https://doi.org/10.17226/10096>

Edmondson, A. C. (2018). *The fearless organization: Creating psychological safety in the workplace for learning, innovation, and growth*. John Wiley & Sons.

Ferlie, E., Ashburner, L., & Pettigrew, A. (1996). *The new public management in action*. Oxford University Press Oxford.

French, W. L., & Bell, C. (1984). *Organization development : behavioral science interventions for organization improvement* (3rd ed.). Prentice-Hall.

Friedmann, J. (1973). *Retracking America : a theory of transactive planning*. Anchor Press Garden City, N.Y.

Furman, C., & Caplan, R. (2007). Applying the Toyota Production System: using a patient safety alert system to reduce error. *Jt Comm J Qual Patient Saf*, *33*(7), 376-386. <https://doi.org/10.1016/s1553-7250(07)33043-2>

Graham, I. D., Logan, J., Harrison, M. B., Straus, S. E., Tetroe, J., Caswell, W., & Robinson, N. (2006). Lost in knowledge translation: time for a map? *J Contin Educ Health Prof*, *26*(1), 13-24. <https://doi.org/10.1002/chp.47>

Greenhalgh, T., Robert, G., Macfarlane, F., Bate, P., & Kyriakidou, O. (2004). Diffusion of innovations in service organizations: systematic review and recommendations. *Milbank Q*, *82*(4), 581-629. <https://doi.org/10.1111/j.0887-378X.2004.00325.x>

Greenwood, R., & Hinings, C. R. (1996). Understanding radical organizational change: Bringing together the old and the new institutionalism. *Academy of management review*, *21*(4), 1022-1054.

Grol, R. P., Bosch, M. C., Hulscher, M. E., Eccles, M. P., & Wensing, M. (2007). Planning and studying improvement in patient care: the use of theoretical perspectives. *Milbank Q*, *85*(1), 93-138. <https://doi.org/10.1111/j.1468-0009.2007.00478.x>

Hammer, M., & Champy, J. (1993). *Reengineering the corporation*. Nicholas Brealey London.

Hines, P., Holweg, M., & Rich, N. (2004). Learning to evolve: a review of contemporary lean thinking. *International journal of operations & production management*, *24*(10), 994-1011.

Holweg, M. (2007). The genealogy of lean production. *Journal of operations management*, *25*(2), 420-437.

Iles, V., & Sutherland, K. (2001). *Managing change in the NHS : organisational change : a review for health care manager, professionals and researchers*. NHS Service Delivery and Organisation London. <http://www.netscc.ac.uk/hsdr/projdetails.php?ref=08-1301-057>

Institute for Healthcare Improvement. (2007). *How to guide. Prevent surgical site infections.* [www.ihi.org/IHI/Topics/PatientSafety/SurgicalSiteInfections/](https://bushare-my.sharepoint.com/personal/mcharns_bu_edu/Documents/Transformation%20Systematic%20Review/Sharing%20MC-LC/www.ihi.org/IHI/Topics/PatientSafety/SurgicalSiteInfections/)

Institute for Healthcare Improvement. (2016). *IHI Model for Improvement*. Retrieved 26 April 2016 from <http://www.ihi.org>

Kegan, R., & Lahey, L. L. (2016). *An everyone culture: Becoming a deliberately developmental organization*. Harvard Business Review Press.

Kirkpatrick, D. L., & Kirkpatrick, J. D. (2006). *Evaluating training programs : the four levels* (3rd ed.). Berrett-Koehler San Francisco, CA.

Kotter, J. P. (2012). *Leading change*. Harvard Business Review Press Boston, Massachusetts.

Kowalski, K., Bradley, K., & Pappas, S. (2006). Nurse retention, leadership, and the Toyota System Model: Building leaders and problem solvers for better patient care. *Nurse Leader*, *4*(6), 46-51.

Liker, J. K. (2004). Toyota Way: 14 Management Principles from the World's Greatest Manufacturer. In: McGraw-Hill Education.

Locke, K. (2011). Field research practice in management and organization studies: Reclaiming its tradition of discovery. *The Academy of Management Annals*, *5*(1), 613-652.

Locke, K., Golden-Biddle, K., & Feldman, M. S. (2008). Perspective—Making doubt generative: Rethinking the role of doubt in the research process. *Organization Science*, *19*(6), 907-918.

Lukas, C. V., Holmes, S. K., Cohen, A. B., Restuccia, J., Cramer, I. E., Shwartz, M., & Charns, M. P. (2007). Transformational change in health care systems: an organizational model. *Health Care Manage Rev*, *32*(4), 309-320. <https://doi.org/10.1097/01.HMR.0000296785.29718.5d>

Martin, R. L. (2009). *The design of business: Why design thinking is the next competitive advantage*. Harvard Business Press.

McCarthy, M. (2006). Can car manufacturing techniques reform health care? *The Lancet*, *367*(9507), 290-291.

National Collaborating Centre for Methods and Tools. (2009). *A model for evidence informed decision making in public health*. <http://www.nccmt.ca/pubs/FactSheet_EIDM_EN_WeB.pdf>

National Health Service National Patient Safety Agency. (2006). *Manchester Patient Safety Framework (MaPSaF): facilitator guidance*. University of Manchester.

Nelson-Peterson, D. L., & Leppa, C. J. (2007). Creating an environment for caring using lean principles of the Virginia Mason Production System. *JONA: The Journal of Nursing Administration*, *37*(6), 287-294.

Nelson, E. C., Batalden, P. B., Huber, T. P., Mohr, J. J., Godfrey, M. M., Headrick, L. A., & Wasson, J. H. (2002). Microsystems in health care: Part 1. Learning from high-performing front-line clinical units. *Jt Comm J Qual Improv*, *28*(9), 472-493. <https://doi.org/10.1016/s1070-3241(02)28051-7>

Nelson, G., Ochocka, J., Griffin, K., & Lord, J. (1998). "Nothing about me, without me": participatory action research with self-help/mutual aid organizations for psychiatric consumer/survivors. *Am J Community Psychol*, *26*(6), 881-912. <https://doi.org/10.1023/a:1022298129812>

Ōno, T., & Bodek, N. (1988). *Toyota production system : beyond large-scale production*. Productivity Press.

Peirce, C. S., & Eisele, C. (1976). *The new elements of mathematics* (Vol. I - IV). Mouton Publishers ; Humanities Press Hague, Atlantic Highlands, N.J.

Peirce, C. S., Hartshorne, C., Weiss, P., & Burks, A. W. (Eds.). (1931-1938). *The Collected Papers of Charles Sanders Peirce* (Vol. 1-8). Harvard University Press.

Peirce, C. S., Houser, N., & Kloesel, C. J. W. (1992). *The essential Peirce : selected philosophical writings. Vol. 1, (1867-1893)*. Indiana University Press Bloomington.

Peirce, C. S., Weiss, P., & Hartshorne, C. (1934). *Collected papers. Vol. 5, Pragmatism and pragmaticism*. Harvard university press Cambridge (Mass.).

Peirce, C. S., Weiss, P., & Hartshorne, C. (1935). *Collected papers of Charles Sanders Peirce. Vol. 6, Scientific metaphysics ; edited by Charles Hartshorne and Paul Weiss*. Harvard University Press Cambridge, Mass.

Perkins, D. D. (1995). Speaking truth to power: Empowerment ideology as social intervention and policy. *American journal of community psychology*, *23*(5), 765-794.

Pettigrew, A., & Whipp, R. (1992). Managing change and corporate performance. In K. Cool, D. Neven, & I. Walter (Eds.), *European industrial restructuring in the 1990s* (pp. 227-265). Macmillan Academic and Professional.

Pettigrew, A. M. (1985). *The awakeing guiant : Continuity and change in imperial chemical industries*. Basil Blackwell Oxford.

Pettigrew, A. M. (1987). Context and action in the transformation of the firm. *Journal of Management Studies*, *24*(6), 649-670.

Pettigrew, A. M. (1992). The character and significance of strategy process research. *Strategic Management Journal*, *13*(S2), 5-16. <https://doi.org/10.1002/smj.4250130903>

Pettigrew, A. M., Ferlie, E., & McKee, L. (1992). *Shaping strategic change : making change in large organizations : the case of the National Health Service*. Sage Publications London.

Pham, H. H., Ginsburg, P. B., McKenzie, K., & Milstein, A. (2007). Redesigning care delivery in response to a high-performance network: the Virginia Mason Medical Center. *Health Aff (Millwood)*, *26*(4), w532-544. <https://doi.org/10.1377/hlthaff.26.4.w532>

Poole, M. S., & Van de Ven, A. H. (2004). Theories of organizational change and innovation processes. In M. S. Poole & A. H. Van de Ven (Eds.), *Handbook of organizational change and innovation* (pp. 374-399). Oxford University Press.

Rappaport, J. (1981). In praise of paradox: A social policy of empowerment over prevention. *American journal of community psychology*, *9*(1), 1-25.

Rogers, E. M. (1995). *Diffusion of innovations* (4th ed.). Free Press New York.

Rogers, E. M. (2003). *Diffusion of innovations* (5th ed.). Free Press New York.

Saegert, S., & Winkel, G. (1996). Paths to community empowerment: Organizing at home. *American journal of community psychology*, *24*, 517-550.

Shani, A., & Pasmore, W. (1985). Organizational inquiry: Towards a new model of the action research process. In D. D. Warrick (Ed.), *Contemporary organization development : current thinking and applications*. Scott, Foresman and Comp.

Stockmeier, C., & Clapper, C. (2009). *The HPI SEC & SSER Patient Safety Measurement System for Healthcare*. Healthcare Performance Improvement, LLC.

Toussaint, J., & Barry, L. (2013). The promise of lean in health care. *Mayo Clin Proc*, *88*(1), 74-82.

US Department of Commerce, N. I. o. S. a. T. (2017). *Baldrige Performance Excellence Program. 2017–2018 Baldrige Excellence Framework (Health Care): A Systems Approach to Improving Your Organization’s Performance*. Retrieved November 6, 2018 from <https://www.nist.gov/baldrige>

Van Maanen, J., Sørensen, J. B., & Mitchell, T. R. (2007). The interplay between theory and method. *Academy of management review*, *32*(4), 1145-1154.

Waterman, H., Tillen, D., Dickson, R., & de Koning, K. (2001). Action research: a systematic review and guidance for assessment. *Health Technol Assess*, *5*(23), iii-157. <https://www.ncbi.nlm.nih.gov/pubmed/11785749>

Weber, D. (2006). Toyota-style management drives Virginia Mason. *Physician executive*, *32*(1), 12.

Womack, J. P., & Jones, D. T. (1996). Beyond Toyota: How to Root Out Waste and Pursue Perfection. *Harvard Business Rev*, *74*, 140-158.

Womack, J. P., Jones, D. T., & Roos, D. (1990). *The machine that changed the world : how Japan's secret weapon in the global auto wars will revolutionize western industry* (1st HarperPerennial ed.). HarperPerennial.

Woodward, E. N., Matthieu, M. M., Uchendu, U. S., Rogal, S., & Kirchner, J. E. (2019). The health equity implementation framework: proposal and preliminary study of hepatitis C virus treatment. *Implementation science*, *14*, 1-18.

Yeich, S., & Levine, R. (1992). Participatory Research's Contribution to a Conceptualization of Empowerment 1. *Journal of Applied Social Psychology*, *22*(24), 1894-1908.

Zimmerman, M. A. (2000). Empowerment theory: Psychological, organizational and community levels of analysis. In *Handbook of community psychology* (pp. 43-63). Springer.

Zimmerman, M. A., & Rappaport, J. (1988). Citizen participation, perceived control, and psychological empowerment. *Am J Community Psychol*, *16*(5), 725-750. <https://doi.org/10.1007/BF00930023>
