# Supplementary material for: Defining and Measuring Organizational Transformation in Health Care: A Systematic Literature Review
Source: Med Care Res Rev. 2025 Aug 13;83(2):71–102. doi: 10.1177/10775587251356130 (PMC12946223; doi:10.1177/10775587251356130)
Supplement: sj-docx-3-mcr-10.1177_10775587251356130 – Supplemental material for Defining and Measuring Organizational Transformation in Health Care: A Systematic Literature Review [file sj-docx-3-mcr-10.1177_10775587251356130.docx]

Part 1.2: 97 English results

“Organizational transformation” OR “Organisational transformation” OR “Organization transformation” OR “Organisation transformation” **Title/Abstract**

Part 1.2 417 English results

“Organizational change” OR “Organisational change” OR “Organization change” OR “Organisation change” **Title Only**

Part 2.1: 1238 English results

“Strategic change” OR “Strategic transformation” OR “Radical change” OR “Radical transformation” OR “Large-system change” OR “Large-system transformation” OR “Transformative change” OR “Transformational change” **Title/Abstract**

**Part 2.2:--Not included—PubMed title/abstract search yielded no results**

“Strategic organisation change” OR “Strategic organization change” OR “Strategic organisational transformation” OR “Strategic organizational transformation” OR “Radical organisation change” OR “Radical organization change” OR “Radical organisational transformation” OR “Radical organizational transformation” OR “Large system organisation change” OR “Large system organization change” OR “Large system organisational transformation” OR “Large system organizational transformation” OR “Transformative organisation change” OR “Transformative organization change” OR “Transformative organisational change” OR “Transformative organizational change” OR “Transformational organisation change” OR “Transformational organization change” OR “Transformational organisational change” OR “Transformational organizational change”

Part 3: 1105 English results

("Culture change" OR "Culture transformation" OR "Cultural change" OR "Cultural transformation") **Title/Abstract** AND (Organization OR Organisation) **Any field**

(((((“Organizational transformation”[Title/Abstract] OR “Organisational transformation”[Title/Abstract] OR “Organization transformation”[Title/Abstract] OR “Organisation transformation”[Title/Abstract]))) OR ((“Organizational change”[Title] OR “Organisational change”[Title] OR “Organization change”[Title] OR “Organisation change”[Title]))) OR ((“Strategic change”[Title/Abstract] OR “Strategic transformation”[Title/Abstract] OR “Radical change”[Title/Abstract] OR “Radical transformation”[Title/Abstract] OR “Large-system change”[Title/Abstract] OR “Large-system transformation”[Title/Abstract] OR “Transformative change”[Title/Abstract] OR “Transformational change”[Title/Abstract]))) OR ((("Culture change" OR "Culture transformation" OR "Cultural change" OR "Cultural transformation") AND (Organization OR Organisation OR Organizational OR Organisational))) AND ((1000/1/1:2022/12/31[pdat]) AND (english[Filter]))

**Filters: English**

3667 total English results (866 new results since last search 9/2019)

('Organizational transformation' or 'Organisational transformation' or 'Organization transformation' or 'Organisation transformation').ab,ti.

OR

('Organizational change' or 'Organisational change' or 'Organization change' or 'Organisation change').ti.

OR

('Strategic change' or 'Strategic transformation' or 'Radical change' or 'Radical transformation' or 'Large system change' or 'Large system transformation' or 'Transformative change' or 'Transformational change').ab,ti.

OR

('Culture change' or 'Culture transformation' or 'Cultural change' or 'Cultural transformation').ab,ti. AND (Organization or Organisation or Organizational or Organisational).af.

2901 total English results (146 new unique references not included in PubMed set or in Embase set from 9/2019)

Limits: Published before 12/31/2022, Pub Types: Article, Article-in-Press, Review
